# Metabolic characterisation of disturbances in the *APOC3*/triglyceride-rich lipoprotein pathway through sample-based recall by genotype

**DOI:** 10.1007/s11306-020-01689-9

**Published:** 2020-06-03

**Authors:** Laura J. Corbin, David A. Hughes, Andrew J. Chetwynd, Amy E. Taylor, Andrew D. Southam, Andris Jankevics, Ralf J. M. Weber, Alix Groom, Warwick B. Dunn, Nicholas J. Timpson

**Affiliations:** 1 MRC Integrative Epidemiology Unit at University of Bristol, Bristol, BS8 2BN UK; 2 Population Health Sciences, Bristol Medical School, University of Bristol, Bristol, BS8 2BN UK; 3 School of Biosciences, University of Birmingham, Edgbaston, Birmingham, B15 2TT UK; 4 Phenome Centre Birmingham, University of Birmingham, Edgbaston, Birmingham, B15 2TT UK; 5 NIHR Biomedical Research Centre at the University Hospitals Bristol NHS Foundation Trust and the University of Bristol, Bristol, BS8 2BN UK; 6 Institute of Metabolism and Systems Research, University of Birmingham, Edgbaston, Birmingham, B15 2TT UK

**Keywords:** *APOC3*, Triglyceride-rich lipoprotein, Genotype, Metabolites, ALSPAC, Recall-by-genotype

## Abstract

**Introduction:**

High plasma triacylglyceride levels are known to be associated with increased risk of atherosclerotic cardiovascular disease. Apolipoprotein C-III (apoC-III) is a key regulator of plasma triacylglyceride levels and is associated with hypertriglyceridemia via a number of pathways. There is consistent evidence for an association of cardiovascular events with blood apoC-III level, with support from human genetic studies of *APOC3* variants. As such, apoC-III has been recognised as a potential therapeutic target for patients with severe hypertriglyceridaemia with one of the most promising apoC-III-targeting drugs, volanesorsen, having recently progressed through Phase III trials.

**Objectives:**

To exploit a rare loss of function variant in *APOC3* (rs138326449) to characterise the potential long-term treatment effects of apoC-III targeting interventions on the metabolome.

**Methods:**

In a recall-by-genotype study, 115 plasma samples were analysed by UHPLC-MS to acquire non-targeted metabolomics data. The study included samples from 57 adolescents and 33 adults. Overall, 12 985 metabolic features were tested for an association with *APOC3* genotype.

**Results:**

144 uniquely annotated metabolites were found to be associated with rs138326449(*APOC3*). The highest proportion of associated metabolites belonged to the acyl-acyl glycerophospholipid and triacylglyceride metabolite classes. In addition to the anticipated (on-target) reduction of metabolites in the triacylglyceride and related classes, carriers of the rare variant exhibited previously unreported increases in levels of a number of metabolites from the acyl-alkyl glycerophospholipid and ceramide classes.

**Conclusion:**

Overall, our results suggest that therapies targeting apoC-III may potentially achieve a broad shift in lipid profile that favours better metabolic health.

**Electronic supplementary material:**

The online version of this article (10.1007/s11306-020-01689-9) contains supplementary material, which is available to authorized users.

## Introduction

The relationship between plasma triacylglyceride (TAG) levels and cardiovascular disease (CVD) has been the focus of much research over the past four decades (Nordestgaard 2016). In that time, a series of population-based cohort studies including several meta-analyses have demonstrated that high plasma TAG levels are associated with increased risk of atherosclerotic CVD (Hokanson and Austin 1996; Sarwar et al. 2007). Alterations in dynamics of triglyceride-rich lipoproteins (TRLs) (i.e. very low-density lipoprotein (VLDL) and chylomicrons), including over-production and/or delayed clearance of VLDL, can lead to high levels of TAGs (hypertriglyceridemia) (Chan et al. 2008). The role of apolipoprotein C-III (apoC-III) in regulating the metabolism of TRLs has been well characterised (Ramms and Gordts 2018). apoC-III is a glycoprotein secreted predominantly by the liver but also by the intestine and is a major component of TRLs but also, to a lesser extent, detectable in high-density lipoproteins (HDL) and low-density lipoprotein (LDL) (Olkkonen et al. 2018). apoC-III is associated with hypertriglyceridemia via a number of pathways, including its action as an inhibitor of lipoprotein lipase (LPL) (the enzyme that metabolises TAGs from TRL to fatty acids and enables their clearance from the circulation) (Chan et al. 2008; Ioanna Gouni-Berthold 2017). There is consistent evidence for an association of cardiovascular events with blood apoC-III level in total plasma or in VLDL and LDL (van Capelleveen et al. 2018; Wyler von Ballmoos et al. 2015) with a suggestion that the pro-atherosclerotic properties of the protein extend beyond its role in increasing TAG levels (Ioanna Gouni-Berthold 2017), including evidence that supports a direct effect of apoC-III on vascular and inflammatory functions (Chan et al. 2008; Kanter et al. 2019; Toth 2016).

Studies in humans have reinforced the potential importance of apoC-III to lipoprotein regulation and CVD risk. The *APOC3* gene sits in a gene cluster with *APOA1, APOA4* and *APOA5* on Chromosome 11. Initially, work was limited to the identification and characterisation of structural variants of *APOC3* present in individuals or small families (reviewed in (van Dijk et al. 2004)). The functional consequences of this variation appears to include effects on lipoprotein metabolism (Liu et al. 2000; von Eckardstein et al. 1991) and insulin responsiveness (Waterworth et al. 2003). More recently, genome-wide association studies (GWAS) conducted on large numbers of individuals have found both common and rare variants in and around this set of genes to be associated with a range of lipid traits (Pollin et al. 2008; Tachmazidou et al. 2013; Willer et al. 2013). Subsequently, a series of large sequencing efforts, both genome-wide (TG and HDL Working Group of the Exome Sequencing Project, National Heart, Lung, and Blood Institute et al. 2014; Timpson et al. 2014) and candidate gene driven (Jørgensen et al. 2014), have contributed a further three apparently cardioprotective *APOC3* variants, including the rare loss of function variant, rs138326449. As well as being informative with respect to apoC-III function in their own right, these studies have delivered a set of genetic variants that can be used in the context of applied genetic epidemiology. For example, stratifying individuals based on *APOC3* genotypes, rather than plasma TAG levels, enables circumvention of confounding factors that affect both plasma TAG levels and CVD, and modelling of life course effects in an approach referred to as Mendelian randomization (MR) (Cohen et al. 2014; Davey Smith and Hemani 2014; Jørgensen et al. 2014; TG and HDL Working Group of the Exome Sequencing Project, National Heart, Lung, and Blood Institute et al. 2014).

Due to its key role in regulating levels of circulating TRLs and supporting genetic evidence, apoC-III has been identified as a potential therapeutic target for patients with severe hypertriglyceridaemia (Olkkonen et al. 2018). Volanesorsen (formerly ISIS 304801) is a second-generation antisense oligonucleotide against *APOC3* mRNA which inhibits apoC-III synthesis and has recently been through Phase III trials (Gaudet et al. 2017; Ionna Gouni-Berthold et al. 2018). Also in the development stage is a monoclonal antibody designed to target lipoprotein bound human apoC-III which mimics the action of a missense variant in *APOC3* (A43T) (Khetarpal et al. 2017). These short-term studies (up to 52 weeks in the case of volanesorsen) appear to show efficacy of apoC-III targeting drugs but critically have not been able to evaluate either the efficacy or safety of targeting apoC-III in the longer term. Therefore, an opportunity exists to provide a detailed characterisation of the long-term effects of apoC-III inhibition for the treatment of hypertriglyceridaemia.

By using recall by genotype (Corbin et al. 2018), a population-based approach derived theoretically from MR, we can offer evidence to strengthen inference from existing observational studies and randomized controlled trials (RCTs). Functional (or functional-linked) genetic variants within *APOC3* can act as proxies for treatment, mimicking the effect of exposure to apoC-III-targeting drugs in a setting analogous to an RCT but, reflecting much longer term (lifetime) exposure. Previously, using a high throughput nuclear magnetic resonance metabolomics platform to quantify over 200 metabolic measures in more than 13,000 individuals, we identified associations between rs138326449(*APOC3*) and VLDL and HDL composition, other cholesterol measures, and fatty acids (Drenos et al. 2016). In the complementary study reported here, we use a recall-by-genotype study design to further probe the downstream consequences of carrying the rare allele at rs138326449(*APOC3*) and in doing so, model the apoC-III-targeting drug response. Using an untargeted ultra high performance liquid chromatography-mass spectrometry (UHPLC-MS) approach to look at and beyond the lipidome to the wider metabolome (Dunn et al. 2011, 2015) we aim to both confirm the likely on-target (intended) treatment effects (i.e. lowering of circulating individual TAGs) but also explore the potential for previously unobserved (off-target) metabolic consequences (that may be linked to positive or negative health outcomes or side effects) of apoC-III targeting interventions.

## Materials and methods

### Study population

The Avon Longitudinal Study of Parents and Children (ALSPAC) is a population-based, prospective birth cohort (Boyd et al. 2013; Fraser et al. 2013). ALSPAC recruited 14,541 pregnant women who were resident in Avon, UK and had expected dates of delivery 1st April 1991 to 31st December 1992. Since then, the health and development of mothers and their children has been followed across the life-course. Further details of the cohort are available in Supplementary Methods (ESM_1) and the study website contains details of all the data that is available through a fully searchable data dictionary and variable search tool (https://www.bristol.ac.uk/alspac/researchers/our-data/). Ethical approval for this study was obtained from the ALSPAC Ethics and Law Committee and the Local Research Ethics Committees. Details of the ethics approvals relevant to the ALSPAC cohort in general can be found on the study website (https://www.bristol.ac.uk/alspac/researchers/research-ethics/) and approvals relevant to this study specifically can be found in Supplementary Methods (ESM_1). Participants have provided informed consent for use of their samples and data.

### Experimental design

#### Sample collection

In this study, we used pre-existing plasma samples collected during routine follow-up clinics. During clinic appointments, blood was drawn into lithium heparin tubes which were centrifuged at 2260* g* for 10 min at 4–5 °C. Heparin plasma was then aliquoted out and stored at − 80 °C. Plasma samples were frozen within 120 min of collection.

#### Sample selection

A recall-by-genotype study design was used to select a subset of stored samples for analysis. Previously, genotyping for the rs138326449 splice variant *APOC3* mutation was performed for all ALSPAC participants (mothers and offspring – referred to herein as ‘young participants’) who had a suitable DNA sample using KASPAR at KBioscience (www.lgcgenomics.com) (Drenos et al. 2016). A list of carriers of the minor ‘A’ allele at rs138326449 was generated and cross-checked against a list of stored samples for these same participants. Where a sample was available for a carrier, samples from two age and sex-matched ‘controls’ (i.e. homozygotes for the major ‘G’ allele) attending the same clinic were identified for inclusion. Where additional samples (from other time points) were available for carriers, these were also selected to be analysed, giving repeated measurements for a subset of participants. In all clinics, participants who were assessed in the morning were instructed to fast overnight and those that attended after 2 pm were instructed to fast for at least six hours prior to their appointment. However, there were nine samples within the mother’s dataset which had to be considered non-fasting since the participant had reported consuming food and/or drink within six hours of blood sampling.

### Ultra high performance liquid chromatography-mass spectrometry (UHPLC-MS)

Plasma samples were extracted applying organic solvents and analysed applying two complementary UHPLC-MS assays applied to study water-soluble metabolites (HILIC assay) and lipid metabolites (C_18_ reversed phase assay). All samples were randomised in their sample preparation and analysis order. A single pooled QC sample was prepared from all biological samples and was applied to quantify and report data quality. Four different datasets were acquired: HILIC positive ion mode (HILIC-POS), HILIC negative ion mode (HILIC-NEG), lipids positive ion mode (LIPIDS-POS) and lipids negative ion mode (LIPIDS-NEG). Raw data processing was performed using XCMS (Smith et al. 2006). Full details of sample preparation, UHPLC-MS analysis and raw data processing can be found in Supplementary Methods (ESM_1).

**Fig. 1:**
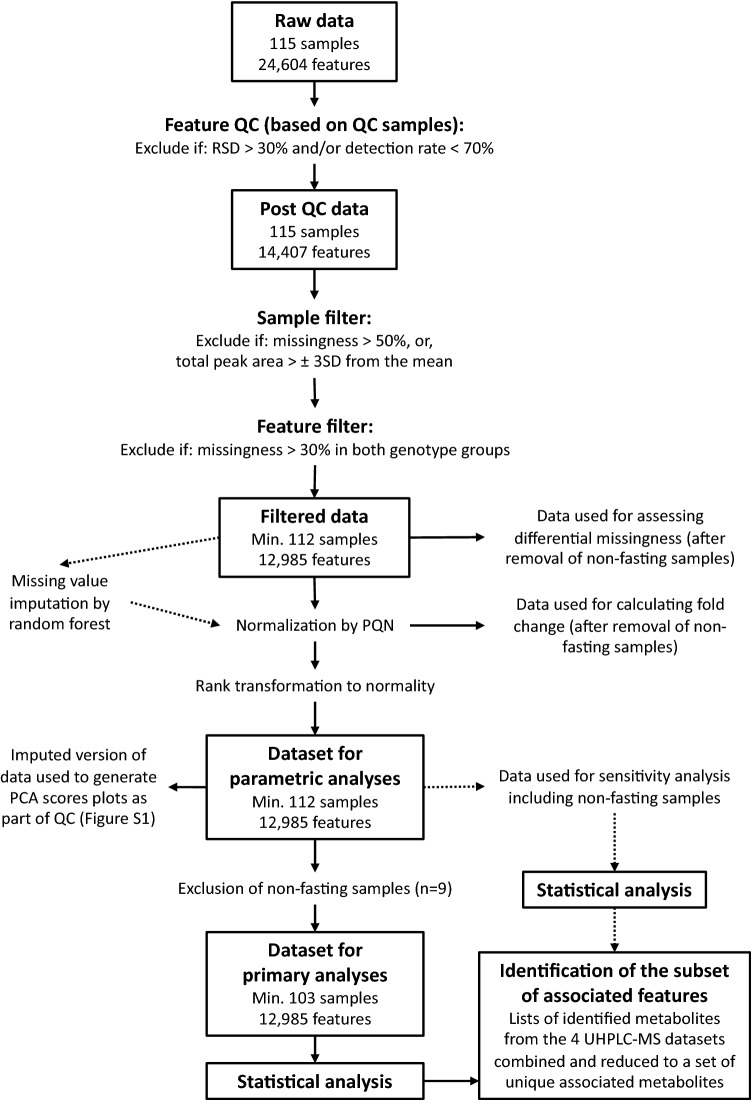
An overview of the data processing pipeline. *PQN* probabilistic quotient normalization, *QC* quality control, *RSD* relative standard deviation, *SD* standard deviations. Dashed lines indicate the flow for sensitivity analyses

An overview of quality control (QC) and data filtering procedures is shown in Fig. 1 and full details provided in Supplementary Methods (ESM_1). In brief, metabolite features with a relative standard deviation (RSD) > 30% and a percentage detection rate < 70% in pooled QC samples were deleted from the dataset.

To increase the quality of the data, filters were applied to the study samples (i.e. excluding pooled QC samples) and features within each of the four UHPLC-MS datasets. Samples with > 50% missing data and/or a total peak area (TPA) greater than three standard deviations above the mean TPA were excluded. After sample exclusion, within-class (carrier vs. control) feature missingness was calculated. Features were excluded from all subsequent analyses if they had > 30% missing data in both classes. Data was then normalised by probabilistic quotient normalisation (PQN) (Frank Dieterle et al. 2006) and rank normal transformed (Aulchenko et al. 2007) prior to statistical analysis.

### Existing lipid measures

In addition to the UHPLC-MS derived metabolic measures, we had available a set of (pre-existing) basic lipid measures assessed by standard laboratory assays (high-density lipoprotein (HDL), low-density lipoprotein (LDL) and total triacylglycerides (TAG)) for a subset of the same individuals and time points. Further details of the measurement of these basic lipid measures can be found in Supplementary Methods (ESM_1). These basic lipid measures were analysed alongside the UHPLC-MS dataset for comparative purposes although the measures themselves are not directly comparable. No transformation was applied to the basic lipid measures prior to analysis.

### Statistical analysis

All statistical analyses described were conducted in R v3.5 or later (R Core Team 2019).

#### Primary analysis

For the primary analysis, we only included samples that could be considered fasting (see above). We tested for an association between the metabolic features (UPLC-MS-derived and existing lipid measures) and rs138326449(*APOC3*) in three datasets: young participants only (n = 21 carriers/42 non-carriers with a mean age of 16 years), mothers only (n = 21 carriers/22 non-carriers, with a mean age of 49 years) and the combined dataset (young participants and mothers) (n = 42 carriers/64 non-carriers). Only samples and features that passed the above QC and filtering steps were analysed. We used either a simple linear model or a mixed linear regression model fitted by maximum likelihood (depending on the number of repeated measures) and including age and sex as fixed effects to test the association between each feature (the dependent variable) and rs138326449(*APOC3*) (for further details see Supplementary Methods (ESM_1)). Features with a Benjamini and Hochberg (BH)-adjusted false discovery rate (FDR) p-value (Benjamini and Hochberg 1995) of < 0.05 in at least one of the three primary analyses (combined, mothers only, young participants only) were considered to be associated with rs138326449(*APOC3*) genotype. For these associated features, the fold change was also calculated as the ratio between the two group means (carrier group mean/non-carrier group mean), using data before rank-transformation was applied. In order to evaluate the variance in metabolite levels explained by the fixed effects (age, sex and genotype), a simple linear model was fitted across all features (making no adjustment for repeated measures or relationships).

Missing values can occur in the data for a number of reasons both technical and biological. One of the main reasons for missing data is that the feature in question is not present in the sample or is only present at very low levels (i.e. its concentration is below the level of detection). In this situation, the linear modelling approach described above would be unable to detect associations between the predictor, rs138326449(*APOC3*) genotype, and presence or absence of the feature. Therefore, a Fisher’s exact test was performed to identify features for which there was a difference in the proportion of missingness between carriers and non-carriers. For this analysis we used the combined dataset (young participants and mothers) (n = 42 carriers/64 non-carriers). Features with a BH-adjusted p-value of *p* < 0.05 were considered to be differentially missing in carriers compared to non-carriers.

#### Sensitivity analyses

Two sensitivity analyses were performed (see Supplementary Methods (ESM_1) for more details). In the first, unlike in the primary analysis, non-fasting samples were retained in the analysis and in the second, an imputed version of the metabolite dataset was analysed.

### Feature identification

We sought to structurally identify all features identified as being associated with rs138326449(*APOC3*) genotype in any one of either the primary or sensitivity analyses. These metabolites were identified applying accurate MS data (and MS/MS fragmentation data, where available) to a confidence level 2 as defined by the Metabolomics Standards Initiative (Sumner et al. 2007). For full details of the identification procedure, see Supplementary Methods (ESM_1). Where multiple features were annotated as the same metabolite (either within or across UHPLC-MS datasets), results for only one feature, that with the strongest association (smallest p-value) with rs138326449(*APOC3*) genotype, were retained. The search for duplicate metabolites was carried out within each of the three datasets (combined, mothers only, young participants only). Results are reported and figures plotted for this set of uniquely annotated features. All lipids are reported according to shorthand notation as has been reported previously (Liebisch et al. 2013).

## Results

The dataset consisted of samples from 57 young participants (‘offspring’) (15 carriers/42 controls) and 33 mothers (11 carriers/ 22 controls), with repeat samples analysed for a subset of carriers (subject to sample availability). In total, 115 samples (51 from carriers and 64 from controls) were analysed by UHPLC-MS. Characteristics of the samples can be found in Table 1; results from a two-sample Wilcoxon rank sum test (Bauer 1972; Hollander and Wolfe 1999) suggested there was no difference in age or BMI between groups, but provided strong evidence for an effect of carrier status on HDL and TAG. There was little evidence for a difference between groups with respect to potential confounders or general indicators of health (Table S1). In total, 12,985 features derived from four UHPLC-MS runs were tested for association with genotype group (for more details see Table S2). Multiple features were detected for each metabolite because of the complexity of ion formation in the electrospray ionisation process and a metabolite can be detected in more than one assay. We estimate the number of metabolites detected to be in the range of 1000–2000 metabolites. After the exclusion of nine non-fasting samples from the mother’s sample set (all from rare variant carriers) for quality control in primary analyses, the number of samples ranged from 103 to 105 (see Fig. 1 for an overview of the data processing pipeline).

**Table 1:** Sample characteristics

	Young participants		Mothers	
	Carriers	Non-carriers	Carriers	Non-carriers
No. of samples (carriers/non-carriers)	21	42	30	22
No. of fasted samples (carriers/non-carriers)^a^	21	42	21	22
No. of unique individuals (carriers/non-carriers)	15	42	11	22
Sex (% females)^b^	47.6%	47.6%	100%	100%
Age (years) at sampling (mean, standard deviation)^b^	16.2 (1.2)	16.2 (1.2)^n^	49.6 (6.0)	47.2 (5.1)^n^
Body mass index (mean, standard deviation)^b^	21.6 (3.2)	22.3 (4.3)^n^	24.8 (3.7)	25.4 (4.5)^n^
HDL mmol/L (mean, standard deviation)^c^	1.62 (0.30)	1.19 (0.25)***	1.93 (0.49)	1.42 (0.25)**
LDL mmol/L (mean, standard deviation)^c^	2.12 (0.45)	1.89 (0.63)^n^	2.56 (0.43)	3.16 (0.80)*
TAG mmol/L (mean, standard deviation)^c^	0.45 (0.06)	0.81 (0.39)***	0.59 (0.14)	1.03 (0.40)**

^a^Non-fasting samples were excluded from the primary analysis

^b^Calculated across all samples (rather than unique individuals)

^c^Using all available data (n = 39 young participants; n = 32 mothers); *HDL* high-density lipoprotein, *LDL* low-density lipoprotein, *TAG* triacylglycerides. A two-sample Wilcoxon rank sum test (Bauer 1972; Hollander and Wolfe 1999) was performed in R v3.6.1 (R Core Team 2019) to test for a difference in quantitative traits between carriers and non-carriers: ^n^ = p > 0.05, *p < 0.05, **p < 0.01, ***p < 0.001

### Over 300 features were identified as associated with rs138326449(*APOC3*)

A total of 311 (2.4%) features showed evidence of association with rs138326449(*APOC3*) in the primary analysis (counting unique features across the three analyses—young participants only, mothers only, and young participants and mothers combined) (Table S3). The highest number of associations was observed in the combined (mothers and young participants) analysis (287), followed by the young participants only analysis (195), with relatively few observed in the mothers only analysis (19). The majority (78.1%) of associations came from features identified in the lipids negative ion mode (LIPIDS-NEG) with relatively few associations coming from the other assays (2.6% from LIPIDS-POS, 19.0% from HILIC-POS, 0.3% from HILIC-NEG). Most features found to be associated in the mothers and/or the young participant’s datasets were also associated in the combined dataset (189 out of 213) (Fig. S1A).

The majority (236 of 287) of the associations seen in the primary analysis (of the combined dataset) replicated in both sensitivity analyses (Fig. S1B). Including non-fasting samples in the analysis resulted in fewer associations whilst using an imputed version of the data increased the number of associated features. Of the basic lipid measures, HDL and TAG were also associated with rs138326449(*APOC3*) in the primary analysis (in all three datasets). Using the simple linear model (including young participants and mothers) we estimated a per ‘A’ allele effect on HDL of 0.51 mmol/l (SE: 0.08, p = 6.85 × 10^−09^) and on total TAG of − 0.40 mmol/l (SE: 0.09, p = 2.02 × 10^−05^).

For the subset of all associated features (n = 311), the percentage variance in standardized individual metabolite levels explained by genotype group ranged from 0.4 to 36.7% (median: 16.2%) as compared to 36.6% in the case of HDL and 23.4% for TAG. This compares to a range of 0.0 to 36.0% (median: 2.1%) explained by age (in years) and 0.0 to 26.9% (median: 0.6%) explained by sex, across all tested features. Estimates of fold change in individual metabolite levels observed in carriers of the rare derived ‘A’ allele relative to controls for the subset of associated features ranged from 0.33 to 1.61 compared to 1.37 for HDL and 0.55 for TAG.

In addition, 53 features were found to have a different degree of missingness in carriers as compared with non-carriers (in the combined dataset). The majority came from the HILIC-POS class (71.7%) with the relatively fewer from the LIPIDS-NEG (22.6%), LIPIDS-POS (3.8%) and HILIC-NEG (1.9%) sets. The vast majority (51/53) had greater levels of missingness in the carrier group than in the non-carrier group, indicating that they occurred at detectable levels less often in the carrier group. Forty-eight of the 53 features identified were not in the list of 311 associated features from the primary (linear model-based) analyses. Therefore, a total of 359 features appear to be influenced by the presence of the rare derived ‘A’ allele at rs138326449.

### Associations were seen predominantly in glycerophospholipids and triacylglycerides

Of the 311 features associated with rs138326449(*APOC3*) in the linear models, 213 could be identified, representing 144 unique metabolites across the three datasets (137 in the combined analysis, 92 in young participants only and 12 in mothers only) (Table S3 and Fig. S2A). Overall, the highest proportion of associated metabolites belong to the acyl-acyl glycerophospholipid (GPL) and TAG metabolite classes (Table 2 and for the full list Table S4). Box and whisker plots showing the distribution of the 213 identified metabolites in carriers versus controls based on normalised data (before rank transformation) in the combined dataset can be found in Fig. S3. 110 unique identified metabolites were found to be common across the primary analysis and the two sensitivity analyses (Fig. S2B). Of the 53 features found to be differentially missing between carriers and non-carriers, 20 were identified (Table S5).

**Table 2:** The distribution across (selected) metabolite classes of identified metabolites from the primary analysis (for full table see Supplementary Table S4)

Metabolite class	No. of associated features (% of total)		
	Combined	Young participants	Mothers
Acyl-acyl GPL	39 (28%)	34 (37%)	0
Triacylglyceride	35 (26%)	7 (8%)	9 (75%)
Ceramide	24 (18%)	20 (22%)	0
Diacylglyceride	14 (10%)	10 (11%)	1 (8%)
Acyl-alkyl GPL	10 (7%)	8 (9%)	0
Sphingolipid	5 (4%)	6 (7%)	0
Total	137	92	12

*GPL* Glycerophospholipid

### A metabolic signature of rs138326449(*APOC3*) carrier status was evident

**Fig. 2:**
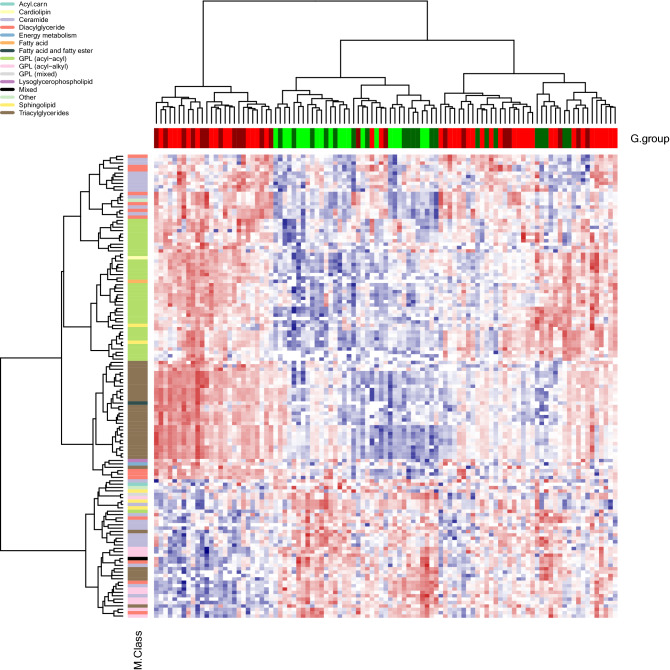
Heatmap showing the 137 unique associated metabolites based on data from the combined dataset residualised on age and sex. *M.Class* = metabolite class (see plot for colour key), *GPL* = glycerophospholipid, *G.group* = genotype group (red = young participants, non-carriers; dark red = mothers, non-carriers; green = young participants, carriers of the ‘A’ allele; dark green = mothers, carriers of the ‘A’ allele (colour figure online)

**Fig. 3:**
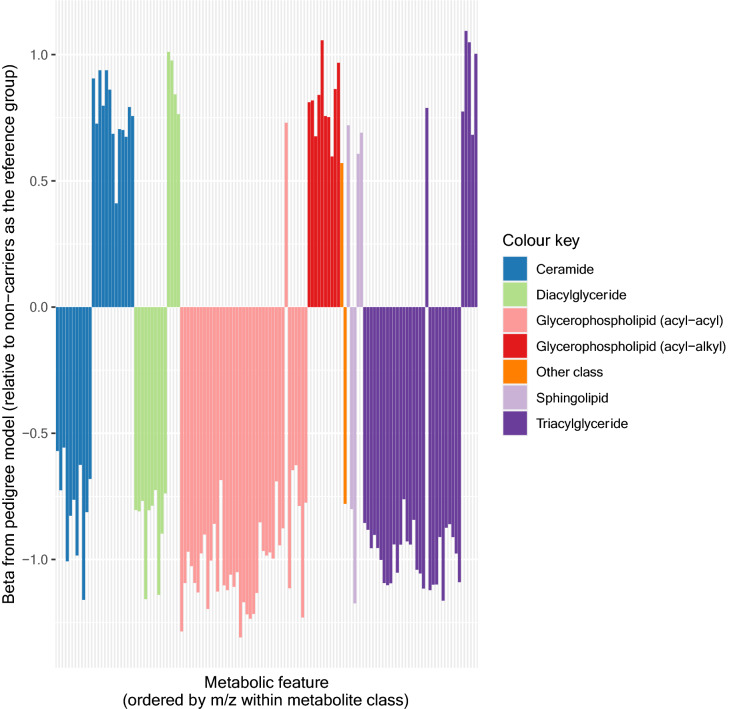
Effect sizes by metabolite class. Effect estimates (betas) taken from linear mixed model with age, sex and pedigree fitted, and using the combined dataset after rank-normal transformation. The same plot ordered by effect estimates (beta) (instead of mass to charge ratio (*m/z*)) can be found in Fig. S5 (colour figure online)

Visualisation of the 137 (identified) unique associated metabolites from the combined analysis in a heatmap with samples and metabolites clustered using Ward’s hierarchical clustering method (Murtagh and Legendre 2014; Ward 1963) as in Fig. 2, suggests broad separation of the samples into three groups—two non-carrier dominated subsets and one carrier dominated subset. Removing metabolites in the TAG class (i.e. those considered to be the primary target of apoC-III lowering therapies) in an exploratory analysis led to improved separation between carriers of the rare allele at rs138326449(*APOC3*) and non-carriers (Fig. S4). Within metabolite classes, the majority of associated metabolites within a class tend to move in the same direction (Fig. S5) with a small number of exceptions that in some cases may relate to molecule size, as proxied by mass to charge ratio (*m/z*) (for example, ceramides) (Fig. 3). Further, within the GPL classes, we observe high correlations between UHPLC-MS-derived measures and those from traditional measures of metabolic health (HDL, LDL, TG) (Fig.’s S6A and S6B).

## Discussion

By exploiting the properties of genetic variation at the level of the population we have provided a comprehensive evaluation of the metabolic consequences of carrying the rare allele at rs138326449(*APOC3*) with a view to informing the therapeutic potential of apoC-III targeting drugs. This work demonstrates how a recall-by-genotype study design can be advantageous given the appropriate conditions—in this case, a relatively rare functional variant (minor allele frequency ~ 0.25% (UK)) in a gene of therapeutic relevance (i.e. a drug target) and a cohort with the necessary genetic data, stored samples and permissions in place to undertake such studies. This work complements existing literature reporting the outcomes of clinical trials of novel apoC-III targeting therapeutic agents, notably, volanesorsen.

A series of Phase I to III trials of this drug and a related drug, AKCEA-APOCIII-L_RX_, have shown decreases of both apoC-III production and TAG concentrations in preclinical models and healthy volunteers (Graham et al. 2013) and in patients with familial chylomicronemia syndrome (FCS) (Gaudet et al. 2014, 2017) and those with hypertriglyceridaemia (Alexander et al. 2019; Gaudet et al. 2015; Ionna Gouni-Berthold et al. 2018). Whilst conclusions from the Phase III trials were that the therapy was generally well-tolerated (indeed, volanesorsen was recently approved in Europe for treatment of FCS (Macchi et al. 2019)), there are two important limitations to these trials in terms of understanding the full effects of such an intervention. Firstly, these trials have not been able to evaluate either the efficacy or safety of targeting apoC-III in the longer term. Long term effects are of particular concern since this kind of drug has the potential to be used chronically following diagnosis with hypertriglyceridemia. Secondly, whilst results from genetic epidemiology suggest the reduction in CVD risk associated with the down-regulation of apoC-III is unlikely to be entirely due to lowering of TRLs, trials to date have measured a relatively small number of outcomes focusing on standard lipoprotein profiles and are therefore not well placed to interrogate alternative pathways. Results here go some way towards addressing this gap in knowledge. The use of samples from both adolescents and adults provide some confidence in terms of the generalisability of our findings and adds further value to this work as a comparator to results of RCTs which are generally restricted in terms of the target population and chronic effects of treatment.

We observed a clear metabolic signature associated with carrying the minor allele at rs138326449(*APOC3*). The list of associated metabolites was dominated by metabolites from the GPL (both acyl-acyl and acyl-alkyl) and TAG classes but also included other lipid classes (diacylglycerides, ceramides, sphingolipids). A detailed summary of our findings, including insight into their potential relevance to health and disease can be found in Table S6 in Supplementary Materials (ESM_1). Overall, our results point to a broad shift in lipid profile that appears to favour better metabolic health in those carrying the minor allele at rs138326449(*APOC3*). We observed not only the anticipated (on-target) reduction of many metabolites in the TAG and acyl-acyl GPL classes in carriers of the rare variant, but also previously unreported increases in levels of a number of other metabolites. Given the current data, it is not possible to evaluate the extent to which each change we see represents a direct effect of the functional change induced by the mutation (‘horizontal pleiotropy’) versus a downstream consequence of that effect (‘vertical pleiotropy’) although it is likely that both of these mechanisms contribute to the profile effects we observe.

The class with the highest proportion of metabolites showing increased concentrations was the acyl-alkyl GPL class. A number of metabolites in this class have previously been associated with positive health effects (see Table S6 for details). In addition, a small number of metabolites in the TAG class showed increased concentrations in rare variant carriers. These six TAGs were amongst the largest TAG molecules identified (as determined by molecular mass (*m/z*)) and had relatively high levels of fatty acid unsaturation (i.e. more fatty acid double bonds). This is interesting in the context of evidence that a distinct pattern exists within TAG subspecies such that the number of carbon atoms and double bonds appear to be relevant with respect to CVD risk (Ho et al. 2016; Rhee et al. 2011; Stegemann et al. 2014; Toledo et al. 2017). Overall, the increase in potentially favourable metabolites may go some way to explaining the previously observed discordance between the size of the cardioprotective effect of *APOC3* and its impact on LDL-cholesterol levels alone (Cohen et al. 2014).

As well as the broad metabolic signature associated with carrying the minor allele at rs138326449(*APOC3*), there are inter-individual differences in metabolic profile. The hierarchical clustering algorithm appeared to differentiate two sub-groups within the non-carrier class, one containing individuals with much higher levels of TAGs and other potentially detrimental molecules than the carrier group and one in which only moderately higher levels were observed. The latter group was more similar to the carrier group overall and included 8 carrier samples (all mother's samples). Factors that could be contributing to the variation in metabolite levels that we see could include other genetic variants and lifestyle differences. Whilst the study was not sufficiently well-powered to enable meaningful comparisons to be made across the two age groups with respect to the associated metabolites, we see a weak correlation between p-value based ranks derived in the mother’s dataset as compared to the young participants (r = 0.24). When combined with the fact that fewer features were associated with carrier status in the mothers as compared to the young participants, one might hypothesise that the effect of the variant is attenuated or at least modified with age, via interaction with environmental variables.

The work presented here is subject to limitations. Due to the low minor allele frequency at rs138326449(*APOC3*), even including all available samples from carriers gave a relatively small total sample size. Therefore, confirmation of our results in other collections would be beneficial. Further limitations relate mainly to the interpretation of our findings rather than the study procedures themselves. We have discussed the possible implications of our findings in the context of apoC-III as a therapeutic target, but genetic models such as this are not a perfect proxy for pharmacologic therapy—any effects on non-apoC-III-related pathways cannot be assessed in this single-gene framework. Furthermore, due to the correlation (linkage disequilibrium) between genetic variants located close together in the genome, the effects we observe may not be entirely attributable to rs138326449(*APOC3*).

In conclusion, we made use of existing samples from an established biobank in a recall-by-genotype framework in order to maximise study power for a given number of samples analysed. The untargeted UHPLC-MS approach used returned results for a large number of metabolic features allowing a more comprehensive evaluation of the impact of rs138326449(*APOC3*) genotype on the global metabolic network of water-soluble and lipid metabolites than has previously been described. Studied in this way, genetic variation represents lifetime exposure to the effects of mutation, which in this case mirrors the action of apoC-III-targeting drugs. Our results suggest therapeutic targeting of apoC-III may result in a positive health effect both through the reduction of harmful lipids but also by increasing levels of beneficial metabolites.

## Electronic supplementary material

Below is the link to the electronic supplementary material.

Supplementary file1 (PDF 2316 kb)

Supplementary file2 (PDF 74 kb)

Supplementary file3 (XLSX 249 kb)
