# Supplementary material for: Metabolic characterisation of disturbances in the APOC3/triglyceride-rich lipoprotein pathway through sample-based recall by genotype
Source: Metabolomics. 2020 Jun 3;16(6):69. doi: 10.1007/s11306-020-01689-9 (PMC7270992; doi:10.1007/s11306-020-01689-9)
Supplement: Supplementary file 1 — Supplementary file1 (PDF 2316 kb) [file 11306_2020_1689_MOESM1_ESM.pdf]

Supplementary Material for:

Metabolic characterisation of disturbances in the *APOC3*/triglyceride-rich lipoprotein pathway through sample-based recall by genotype

Corbin *et al.*

Correspondence: Prof. Nicholas Timpson (n.j.timpson@bristol.ac.uk)

SUPPLEMENTARY METHODS

Sample selection

Young participants

In the case of offspring (referred to herein as 'young participants'), metabolic measures were obtained from plasma taken at follow-up clinic assessments at the approximate ages of 15 and 17 years. Where a sample was available for a carrier, samples from two matched 'controls' (i.e. homozygotes for the major 'G' allele) were identified. Controls were matched for sex and age, such that the two sex-matched participants closest in age to the carrier (and with samples available) were selected for inclusion in the analysis. In cases where samples for a carrier individual were available from both clinics (age 15 and age 17), both samples were analysed giving repeated measurements for a subset of participants.

Mothers

In the case of mothers, metabolic measures were obtained from plasma taken at the first of four follow-up clinic assessments that have been conducted in this population to date. Where a sample was available for a carrier in clinic 1, two matched 'control' (i.e. homozygotes for the major 'G' allele) samples were identified. Controls were matched for age, such that the participants closest in age to the carrier at clinic 1 (with a sample available) were selected for inclusion in the analysis. In addition, where samples for a carrier individual were available from any of the subsequent clinics (mother's clinics 2-4), all available samples (one per clinic) were analysed giving repeated measurements for a subset of participants. The median age of mothers who attended these clinics was 48 years at clinic 1, 50 years at clinic 2, 53 years at clinic 3 and 54 years at clinic 4.

Ultra High Performance Liquid Chromatography-Mass Spectrometry Procedure

Sample preparation

Plasma samples were extracted to separate low molecular weight metabolites from other biochemicals including proteins, RNA and DNA. For the analysis of water-soluble metabolites in a HILIC assay, 180 µL of acetonitrile (LC-MS grade, LiChrosolv, Merck) was added to 60 µL of plasma followed by vortex mixing (15 seconds), centrifugation (13,000 ×g, 15 min) and transfer of the clear supernatant to a glass LC autosampler vial (VI-04-12-02RVG 300µl Plastic, Chromatography Direct, UK). For the analysis of lipid metabolites, 180 µL of isopropyl alcohol, IPA (LC-MS grade, LiChrosolv, Merck) was added to 60 µL of plasma followed by

vortex mixing (15 seconds), centrifugation (13,000×g, 15 minutes) and transfer of the clear supernatant to a glass LC autosampler vial (VI-04-12-02RVG 300µl Plastic, Chromatography Direct, UK). A single pooled QC sample was prepared by combining 25 µL aliquots of all biological samples where adequate sample volume remained and vortex mixing (5 minutes). Aliquots (60 µL) of the pooled QC sample were extracted as defined above.

Ultra High Performance Liquid Chromatography-Mass Spectrometry

The samples were analysed applying two Ultra Performance Liquid Chromatography-Mass Spectrometry (UHPLC-MS) methods using a Dionex UltiMate 3000 Rapid Separation LC system (Thermo Fisher Scientific, MA, USA) coupled with and electrospray Q Exactive Focus mass spectrometer (Thermo Fisher Scientific, MA, USA). Polar extracts were analysed on a Accucore-150-Amide-HILIC column (100 x 2.1 mm, 2.6 µm, Thermo Fisher Scientific, MA, USA). Mobile phase A consisted of 10 mM ammonium formate and 0.1% formic acid in 95% acetonitrile/water and mobile phase B consisted of 10 mM ammonium formate and 0.1% formic acid in 50% acetonitrile/water. Flow rate was set for 0.50 $mL{\cdot}min^{-1}$ with the following gradient: t=0.0, 1% B; t=1.0, 1% B; t=3.0, 15% B; t=6.0, 50% B; t=9.0, 95% B; t=10.0, 95% B; t=10.5, 1% B; t=14.0, 1% B, all changes were linear with curve = 5. The column temperature was set to 35 °C and the injection volume was 2 µL. Data were acquired in positive and negative ionisation modes separately within the mass range of 70 – 1050 m/z at resolution 70,000 (FWHM at m/z 200). Ion source parameters were set as follows: Sheath gas = 53 arbitrary units, Aux gas = 14 arbitrary units, Sweep gas = 3 arbitrary units, Spray Voltage = 3.5kV, Capillary temp. = 269 °C, Aux gas heater temp. = 438 °C. Data dependent MS2 in 'Discovery mode' was used for the MS/MS spectra acquisition using following settings: resolution = 17,500 (FWHM at m/z 200); Isolation width = 3.0 m/z; stepped normalised collision energies (stepped NCE) = 25, 60, 100%. Spectra were acquired in three different mass ranges: 50 – 200 m/z; 200 – 400 m/z; 400 – 1000 m/z. Non-polar extracts were analysed on Hypersil GOLD column (100 x 2.1mm, 1.9 µm; Thermo Fisher Scientific, MA, USA). Mobile phase A consisted of 10 mM ammonium formate and 0.1% formic acid in 60% acetonitrile/water and mobile phase B consisted of 10 mM ammonium formate and 0.1% formic acid in 90% propan-2-ol/water. Flow rate was set for 0.40 mL.min-1 with the following gradient: t=0.0, 20% B; t=0.5, 20% B, t=8.5, 100% B; t=9.5, 100% B; t=11.5, 20% B; t=14.0, 20% B, all changes were linear with curve = 5. The column temperature was set to 55 °C and the injection volume was 2µL. Data were acquired in positive and negative ionisation mode separately within the mass range of 150 – 2000 m/z at resolution 70,000 (FWHM at m/z 200). Ion source parameters were

set as follows: Sheath gas = 50 arbitrary units, Aux gas = 13 arbitrary units, Sweep gas = 3 arbitrary units, Spray Voltage = 3.5kV, Capillary temp. = 263 °C, Aux gas heater temp. = 425 °C. Data dependent MS2 in 'Discovery mode' was used for the MS/MS spectra acquisition using following settings: resolution = 17,500 (FWHM at m/z 200); Isolation width = 3.0 m/z; stepped normalised collision energies (stepped NCE) = 20, 50, 80%. Spectra were acquired in three different mass ranges: 200 – 400 m/z; 400 – 700 m/z; 700 – 1500 m/z.

A Thermo ExactiveTune 2.8 SP1 build 2806 was used as an instrument control software in both cases and data were acquired in profile mode. Quality control (QC) samples were analysed as the first ten injections and then every seventh injection with two QC samples at the end of the analytical batch. Two blank samples were analysed, the first as the 6th injection and then at the end of each batch.

Raw data processing

Raw data acquired in each analytical batch were converted from the instrument-specific format to the mzML file format applying the open access ProteoWizard software (Chambers et al. 2012). Deconvolution was performed with XCMS software according to the following settings of min. peak width (4 for HILIC and 6 for LIPIDS); max. peak width (30); ppm (12 for HILIC and 14 for LIPIDS); mzdiff (0.001); bw (0.25); mzwid (0.01) (Smith et al. 2006). A data matrix of metabolite features (*m/z*-retention time pairs) vs. samples was constructed with peak areas provided where the metabolite feature was detected for each sample. Data for the first 8 QC samples were removed from the dataset. Putative annotation of metabolites or metabolite groups was performed by applying the PUTMEDID-LCMS workflows operating in the Taverna workflow environment (Brown et al. 2011). We applied 5 ppm mass error and a retention time range of 2 s in feature grouping and molecular formula and metabolite matching. Because different metabolites can be detected with the same accurate m/z (for example, isomers with the same molecular formula), multiple annotations could be observed for a single detected metabolite feature. Also, a single metabolite could be detected as multiple molecules, particularly as a different type of ion (e.g., protonated and sodiated ions). Throughout this article, the term "metabolite" refers to either single metabolites or groups of molecules with the same retention time and the same accurate m/z. All molecules were annotated according to guidelines for reporting of chemical analysis results, specifically to Metabolomics Standards Initiative level 2 (Sumner et al. 2007).

Assessment of data quality

The data for pooled QC samples were applied to perform QC filtering. Data matrices were corrected for run-order drift in intensity using the Quality Control-Robust Spline Correction (QC-RSC) algorithm (Kirwan et al. 2013) (https://github.com/computational-metabolomics/sbcms). For each metabolite feature detected QC samples 1-8 were removed and the relative standard deviation (RSD) and percentage detection rate were calculated. Metabolite features with a relative standard deviation (RSD) > 30% and a percentage detection rate < 70% were removed from the dataset. Details of the numbers of features before ("Raw data") and after this process ("Post-QC data") can be found in **Table S2**.

Post-raw data processing workflow

A series of procedures were applied to the data derived from the processing steps described above in preparation for the statistical analysis of the data, as described in **Fig. 1**. All procedures were carried out in R v3.5 or later (R Core Team 2019). Each of the four datasets (HILIC-POS, HILIC-NEG, LIPIDS-POS and LIPIDS-NEG) was processed separately as follows. To increase the quality of the data, two filters were applied to the study samples (i.e. not including QC samples) and one to the features. Firstly, samples were excluded from all subsequent analyses if they had more than 50% missing data across the set of features. This resulted in one sample exclusion from the HILIC-POS dataset. Secondly, total peak area was calculated for each sample and samples whose calculated peak area fell outside three standard deviations from the mean were excluded. This resulted in two sample exclusions from the HILIC-NEG dataset, three from the HILIC-POS dataset, one from the LIPIDS-NEG dataset and three from the LIPIDS-POS dataset. After sample exclusion, within-class (carrier *vs.* control) feature missingness was calculated. Features were excluded from all subsequent analyses if they had >30% missing data in both classes. Details of the numbers of features and after this process ("Post-filtering data") can be found in **Table S2**.

As is typical in metabolite data of this nature, levels of feature missingness were relatively high (up to 52.6% missing) with the majority of missing data points likely due to very low levels of

the metabolite, i.e. below the level of detection. Therefore, an iterative imputation method based on a random forest was used to impute missing values in the dataset. The R package missForest (Stekhoven and Buhlmann 2012) was used with its default settings. Both the unimputed and the imputed versions of the data were taken forward into the analysis with results from the former representing the primary analysis and those from the latter a sensitivity analysis included to assess the likely impact of missing data on our primary analysis.

In preparing the data for parametric analyses, the next step was normalisation. Data were normalised across samples using probabilistic quotient normalisation (Frank Dieterle et al. 2006) (PQN) implemented using the normalisation function in the KODAMA R package (Cacciatore et al. 2016; Eisen et al. 1998; Frank Dieterle et al. 2006). The median value across all available QC samples in the dataset was used as the reference sample for PQN. Following normalisation, the QC samples were removed from the dataset. Exploration of the data revealed the distributions of 53-56% (range across the four datasets) of features to be non-normal based on a Shapiro-Wilk test of normality (w>0.95) (J. P. Royston 1982a, 1982b; P. Royston 1995). Therefore, a rank-transformation to normality (Aulchenko et al. 2007) was applied across all features in preparation for statistical analysis.

There is evidence that levels of at least some metabolites can be influenced by fasting time (Sedlmeier et al. 2018) and for this reason, we conducted our primary analysis only on fasted samples. However, this resulted in the exclusion of nine samples from the analysis, all from rare variant carriers. Therefore, we conducted a sensitivity analysis in which the primary analysis of the combined dataset was rerun as described above but with no sample exclusions being made based on the fasting/non-fasting status of the samples (n=51 carriers/64 non-carriers).

The imputed version of the data was used to assess the technical variability (measured by the replicate analysis of a pooled QC sample) and biological variability as part of the quality control process. Data were normalised and rank-transformed as described above but with QC samples retained. A principal components analysis (PCA) was then performed using the prcomp() function in R. PCA score plots are shown for the four datasets (HILIC-POS, HILIC-NEG, LIPIDS-POS and LIPIDS-NEG) in **Fig. S7**.

Existing lipid measures

Children: All assays were completed at Professor Naveed Sattar's laboratory at the University of Glasgow. Plasma lipids (total cholesterol, triacylglycerides (TAG) and high-density lipoprotein (HDL) cholesterol) were performed by modification of the standard Lipid Research Clinics Protocol using enzymatic reagents for lipid determination. Low-density lipoprotein (LDL) cholesterol was calculated using the Friedwald equation: LDL = total cholesterol - (HDL + (TG*0.45)).

Mothers: Cholesterol, triacylglycerides (TAG) and high-density lipoprotein (HDL) cholesterol enzymatic colorimetic assays were performed by the staff of the Routine Lipids Section of the Biochemistry Department of Glasgow Royal Infirmary using a Hitachi Modular P Analyser. Kits were supplied by Roche Diagnostics Gmbh (Cholesterol kit (CHOD-PAP) cat no. 1491458; Triglyceride kit (GPO-PAP) cat no. 1730711; High-density lipoprotein cholesterol kit (2nd Generation) cat no. 3045935). Low-density lipoprotein (LDL) cholesterol results were calculated using the Friedwald Equation: LDL = total cholesterol - (HDL cholesterol + (TG/2.19)).

Statistical analysis

Models fitted

In the young participants only and mothers only analyses we used either a simple linear multivariate model or a mixed linear multivariate regression model fit by maximum likelihood to test the association between each feature (the dependent variable) and rs138326449(*APOC3*). Only samples with non-missing data for the feature being tested were included in the analysis of that same feature (hence the actual sample size varies by feature). If, after excluding samples with missing data, less than five repeated measures remained in the dataset, a simple linear model was used (fitted using the R function lm()), otherwise a mixed linear regression model was fitted with individual included as a random effect in order to take account of repeated measures (lmer()). In order to combine all data (young participants and mothers) into a single model, we extended the mixed linear regression model to incorporate the effects of pedigree using the R package 'pedigreemm' (Vazquez et al. 2010). In this way we were able to include both young participants' and mothers' data in the same model whilst accounting for mother-child relationships and repeated measures within the sample set. All models were fitted with age and sex (except in mothers only analyses) fitted as fixed effects.

The association between each feature and rs138326449(*APOC3*) was tested by performing a likelihood ratio test of nested models, comparing models with and without class status (carrier *vs.* control) fitted as a fixed effect. Resulting p-values were then adjusted to take account of multiple testing using the false discovery rate-based method of Benjamini and Hochberg (BH) (Benjamini and Hochberg 1995).

Feature identification

PUTMEDID_LCMS (Brown et al. 2011) was applied to annotate metabolites using accurate mass-to-charge (*m/z*) data using KEGG, the Human Metabolome Database and LipidMaps as reference databases containing all known metabolites. LipidSearch (Peake et al. 2015) was applied to annotate metabolites using accurate *m/z* MS/MS data with matching of experimental MS/MS data to theoretical MS/MS data. To obtain greater confidence in the metabolite annotation, experimental MS/MS fragmentation data was also compared to the reference mass spectral libraries mzCloud (www.mzcloud.org) and the in-silico mass spectral library using the LipidSearch software (Peake et al. 2015). The expected retention time ranges, derived from authentic chemical standards, were applied to remove false annotations related to lipid metabolites.

Data visualisation

Venn diagrams were generated using the R package VennDiagram (Chen and Boutros 2011) to show the overlap between associated features identified firstly, in the three primary analyses and, secondly, in the primary analysis compared to the sensitivity analyses. Heatmaps were generated using the R package heatmap3 (Zhao et al. 2014). Input data for the heatmaps was the rank-transformed data for the subset of associated and identified features, residualised on age and sex. Dendrograms were constructed in R v3.5 or later (R Core Team 2019) ('stats' package) based on (Euclidean) distance matrices and subsequent clustering based on Ward's method (Ward 1963), the Ward2 algorithm (Murtagh and Legendre 2014). All other plots were created in ggplot2 (Wickham 2016).

ALSPAC Cohort description

Description of study numbers

ALSPAC recruited 14,541 pregnant women resident in Avon, UK with expected dates of delivery 1st April 1991 to 31st December 1992. 14,541 is the initial number of pregnancies for which the mother enrolled in the ALSPAC study and had either returned at least one questionnaire or attended a "Children in Focus" clinic by 19/07/99. Of these initial pregnancies, there was a total of 14,676 foetuses, resulting in 14,062 live births and 13,988 children who were alive at 1 year of age.

When the oldest children were approximately 7 years of age, an attempt was made to bolster the initial sample with eligible cases who had failed to join the study originally. As a result, when considering variables collected from the age of seven onwards (and potentially abstracted from obstetric notes) there are data available for more than the 14,541 pregnancies mentioned above.

The number of **new pregnancies** not in the initial sample (known as Phase I enrolment) that are currently represented on the built files and reflecting enrolment status at the age of 18 is 706 (452 and 254 recruited during Phases II and III respectively), resulting in an additional 713 children being enrolled. The phases of enrolment are described in more detail in the cohort profile paper.

The total sample size for analyses using any data collected after the age of seven is therefore 15,454 pregnancies, resulting in 15,589 foetuses. Of this **total sample** of 15,656 foetuses, 14,973 were **live births** and 14,899 were **alive at 1 year of age**.

A 10% sample of the ALSPAC cohort, known as the **Children in Focus (CiF) group**, attended clinics at the University of Bristol at various time intervals between 4 to 61 months of age. The CiF group were chosen at random from the last 6 months of ALSPAC births (1432 families attended at least one clinic). Excluded were those mothers who had moved out of the area or were lost to follow-up, and those partaking in another study of infant development in Avon.

Data management

Study data were collected and managed using REDCap electronic data capture tools hosted at University of Bristol (Harris et al. 2009). REDCap (Research Electronic Data Capture) is a secure, web-based application designed to support data capture for research studies, providing 1) an intuitive interface for validated data entry; 2) audit trails for tracking data manipulation

and export procedures; 3) automated export procedures for seamless data downloads to common statistical packages; and 4) procedures for importing data from external sources.

Details of ethics approvals

Consent for biological samples has been collected in accordance with the Human Tissue Act (2004). Informed consent for the use of data collected via questionnaires and clinics was obtained from participants following the recommendations of the ALSPAC Ethics and Law Committee at the time. Details of ethics approvals relevant to this study:

- 15-year clinic: Central & South Bristol Research Ethics Committee (UBHT): 06/Q2006/53 Avon Longitudinal Study of Parents and Children (ALSPAC), Hands on Assessments: Teen Focus 3 (Focus 15+) (7th August 2006) (Confirmed 15th September 2006).
- 17-year clinic: North Somerset & South Bristol Research Ethics Committee: 08/H0106/9 Avon Longitudinal Study of Parents and Children (ALSPAC), Hands on Assessments: Teen Focus 4 (Focus 17+) (18th November 2008).
- Mothers clinic 1: North Somerset & South Bristol Research Ethics Committee: 08/H0106/96 Avon Longitudinal Study of Parents and Children (ALSPAC); Focus on Mothers (2nd September 2008, Confirmed 8th September 2008).
- Mothers clinics 2&3: National Research Ethics Service Committee South West – Central Bristol: 11/SW/0110 Avon Longitudinal Study of Parents and Children (ALSPAC); Focus on Mothers 2 and 3 (1st June 2011, Confirmed 20th June 2011).
- Mothers clinic 4: National Research Ethics Service Committee South West – Central Bristol: 11/SW/0110 Avon Longitudinal Study of Parents and Children (ALSPAC) - amendment; Focus on Mothers 4 (6th January 2014, Confirmed 21st February 2014).

SUPPLEMENTARY FIGURES

Fig. S1A. Number and overlap of associated features across the three primary analyses

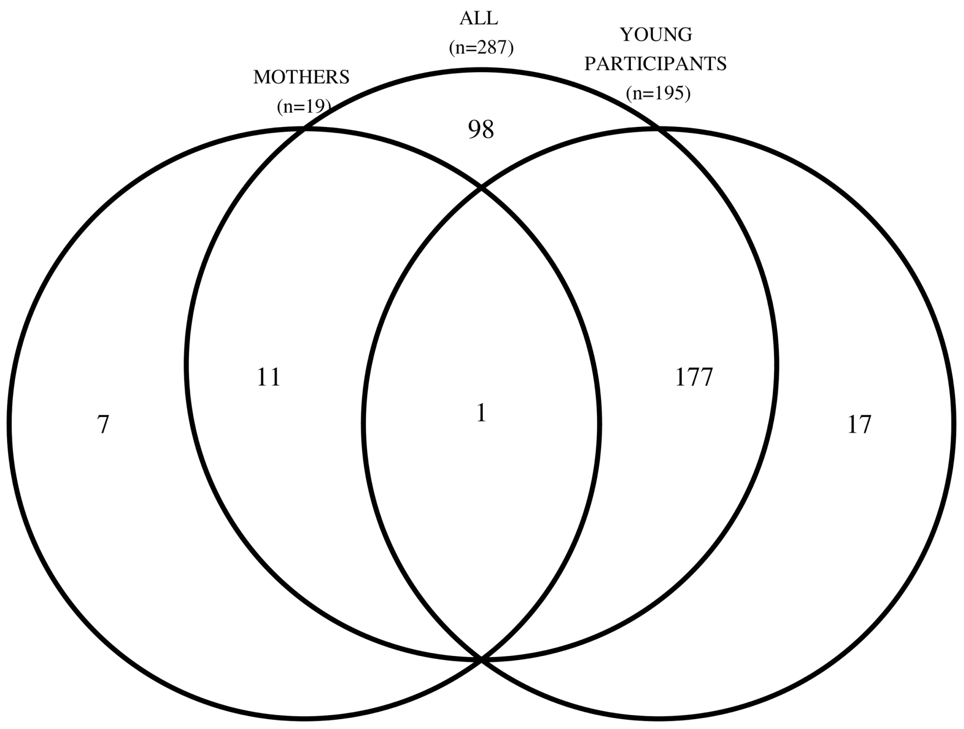

Fig. S1B. Number and overlap of associated features across the primary analysis and two sensitivity analyses

MAIN = primary analysis (as described in methods and presented in main manuscript); MVI = analysis based on missing value imputed data; NON-FASTING = analysis including 9 non-fasting samples. In all cases analysis conducted on young participants and mothers combined.

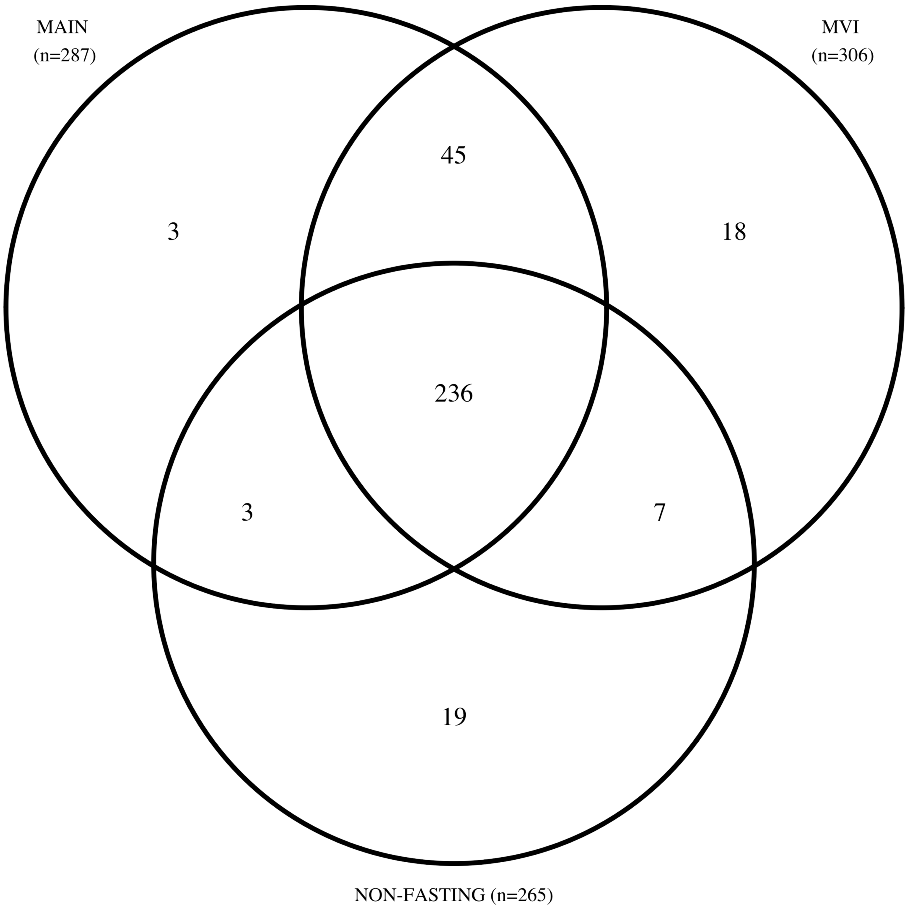

Fig. S2A. Number and overlap of unique associated and identified metabolites across the three primary analyses

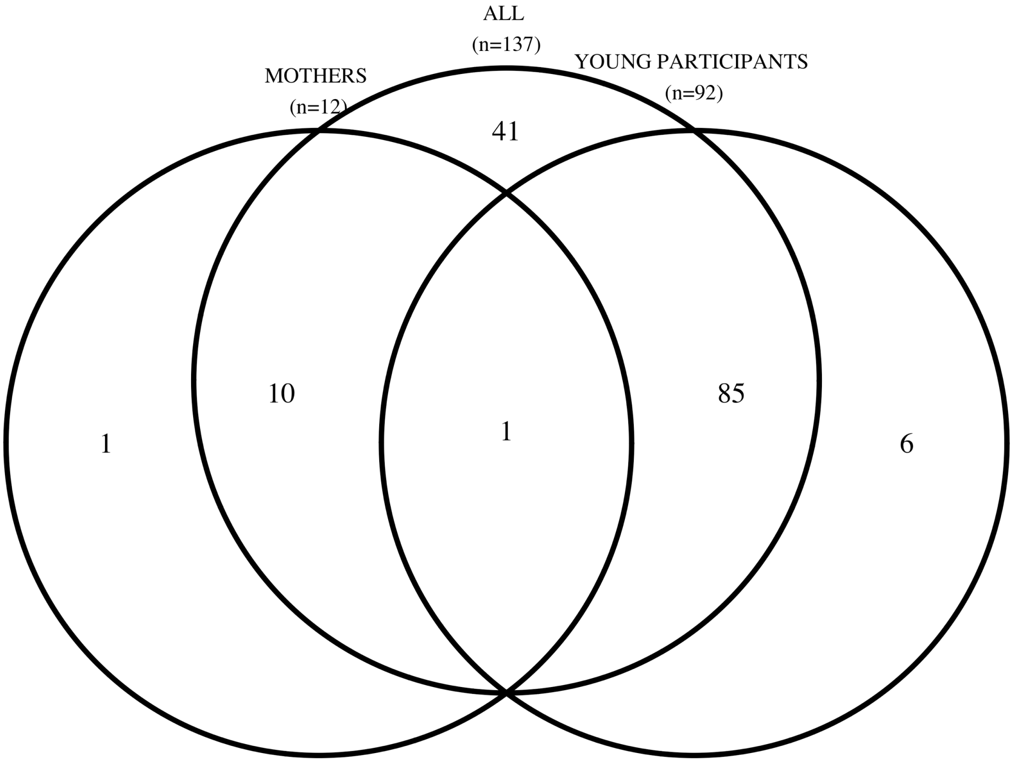

Fig. S2B. Number and overlap of unique associated and identified metabolites across the primary analysis and two sensitivity analyses

MAIN = primary analysis (as described in methods and presented in main manuscript); MVI = analysis based on missing value imputed data; NON-FASTING = analysis including 9 non-fasting samples. In all cases analysis conducted on young participants and mothers combined.

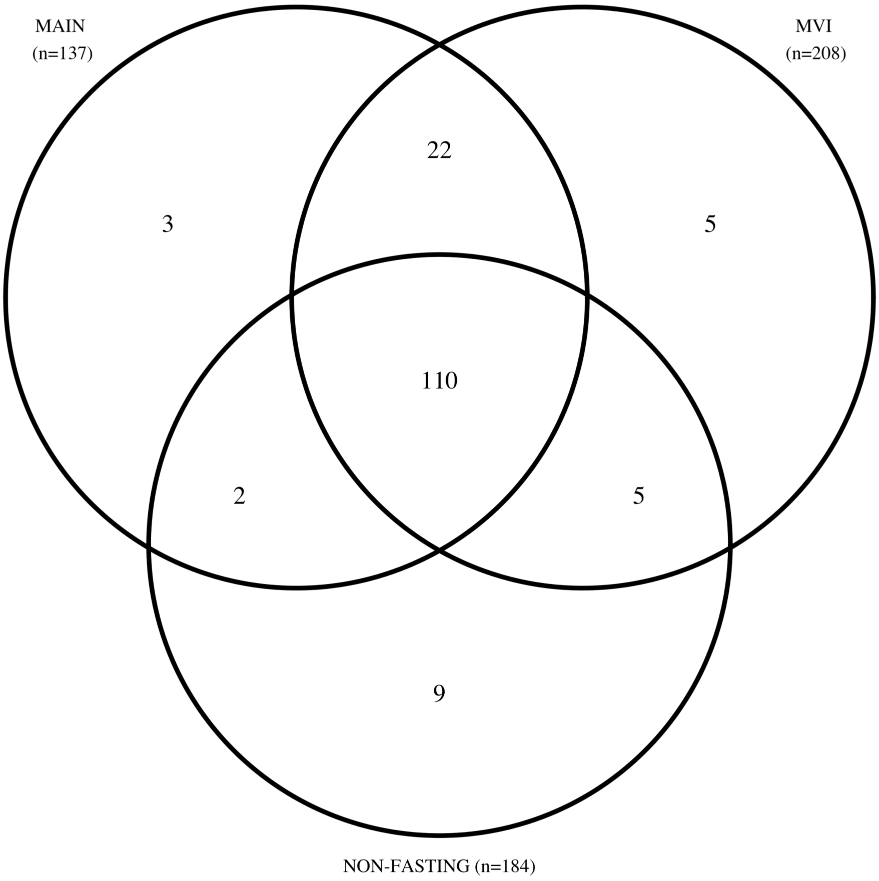

Fig. S3. Box and whisker plots showing the distribution of the 213 identified metabolites in carriers versus controls based on normalised data (pre-rank transformation) across all samples

SEE ADDITIONAL SUPPLEMENTARY PDF FILE

Fig. S4. Heatmap showing 102 of the unique associated metabolites (following exclusion of metabolites in the triacylglyceride class) based on data from the combined dataset residualised on age and sex.

M.Class = metabolite class (see plot for colour key); GPL = glycerophospholipid; G.group = genotype group (red = young participants, non-carriers; dark red = mothers, non-carriers; green = young participants, carriers of the 'A' allele; dark green = mothers, carriers of the 'A' allele

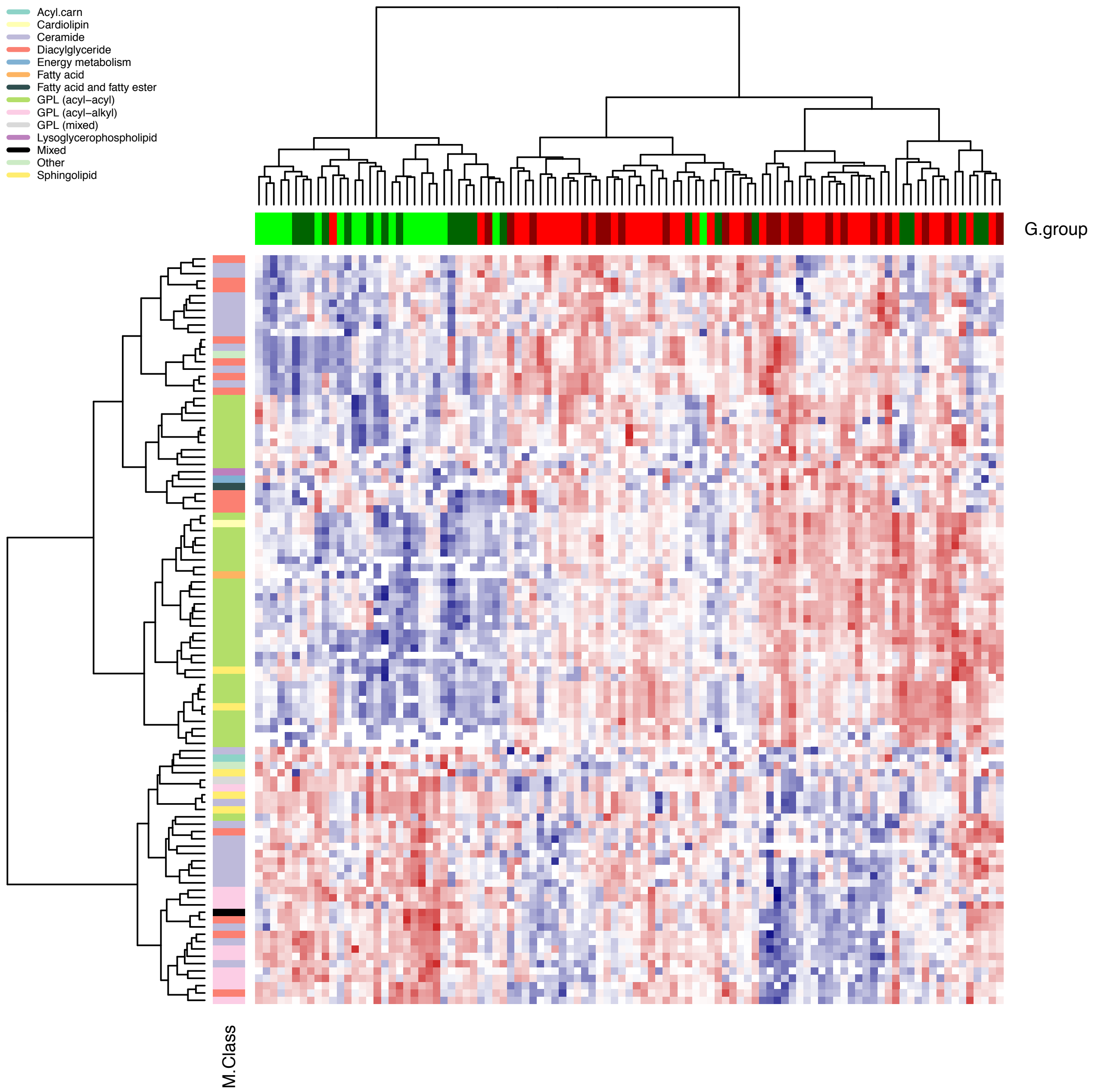

Fig. S5. Effect sizes by metabolite class.

Betas taken from linear mixed model with pedigree fitted and using the combined dataset after rank normal transformation.

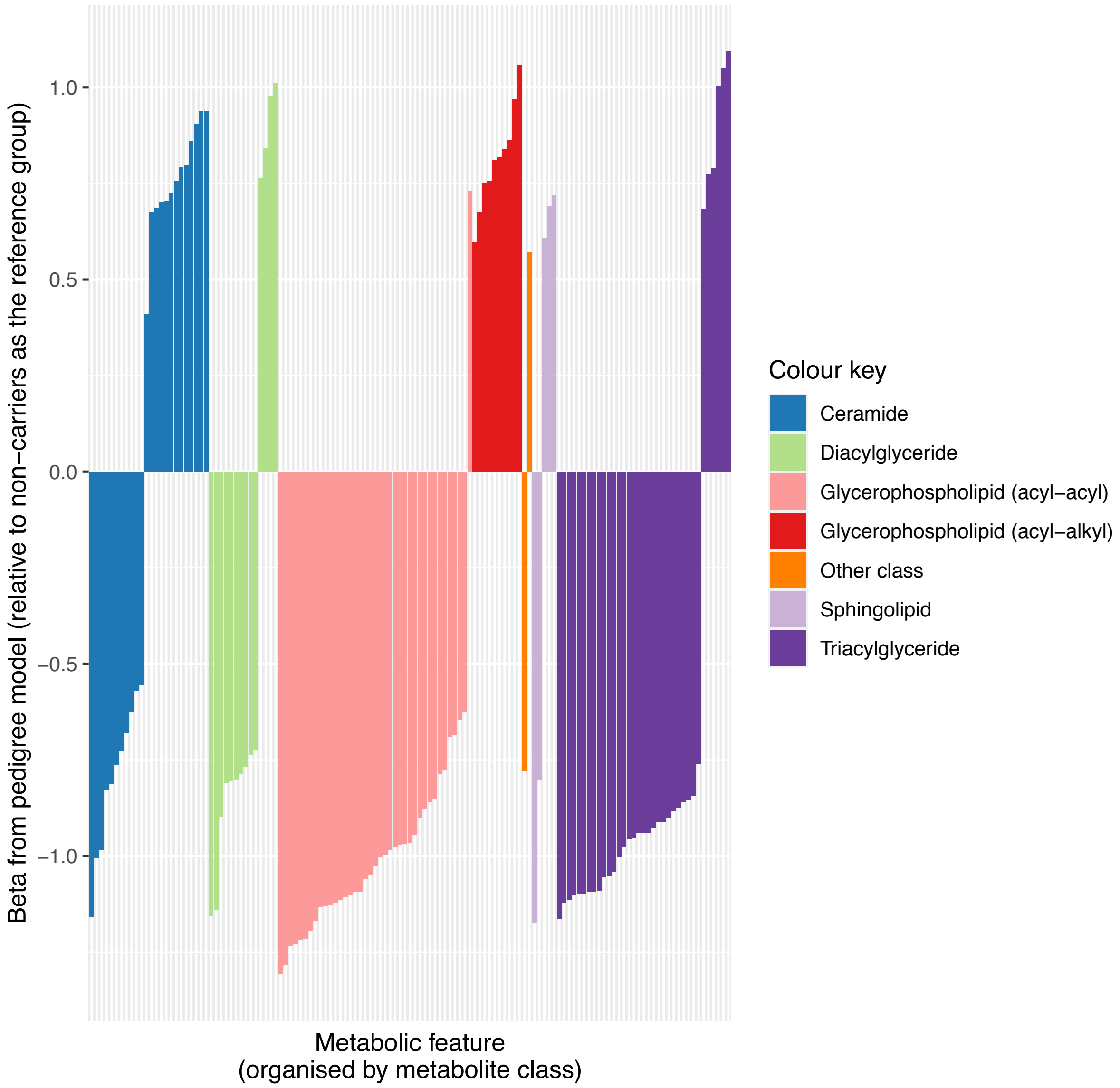

Fig. S6A. Correlation heatmap showing the relationship between associated metabolites in the glycerophospholipid (acyl-alkyl) class and traditional measures of metabolic health (Total TAG, LDL and HDL).

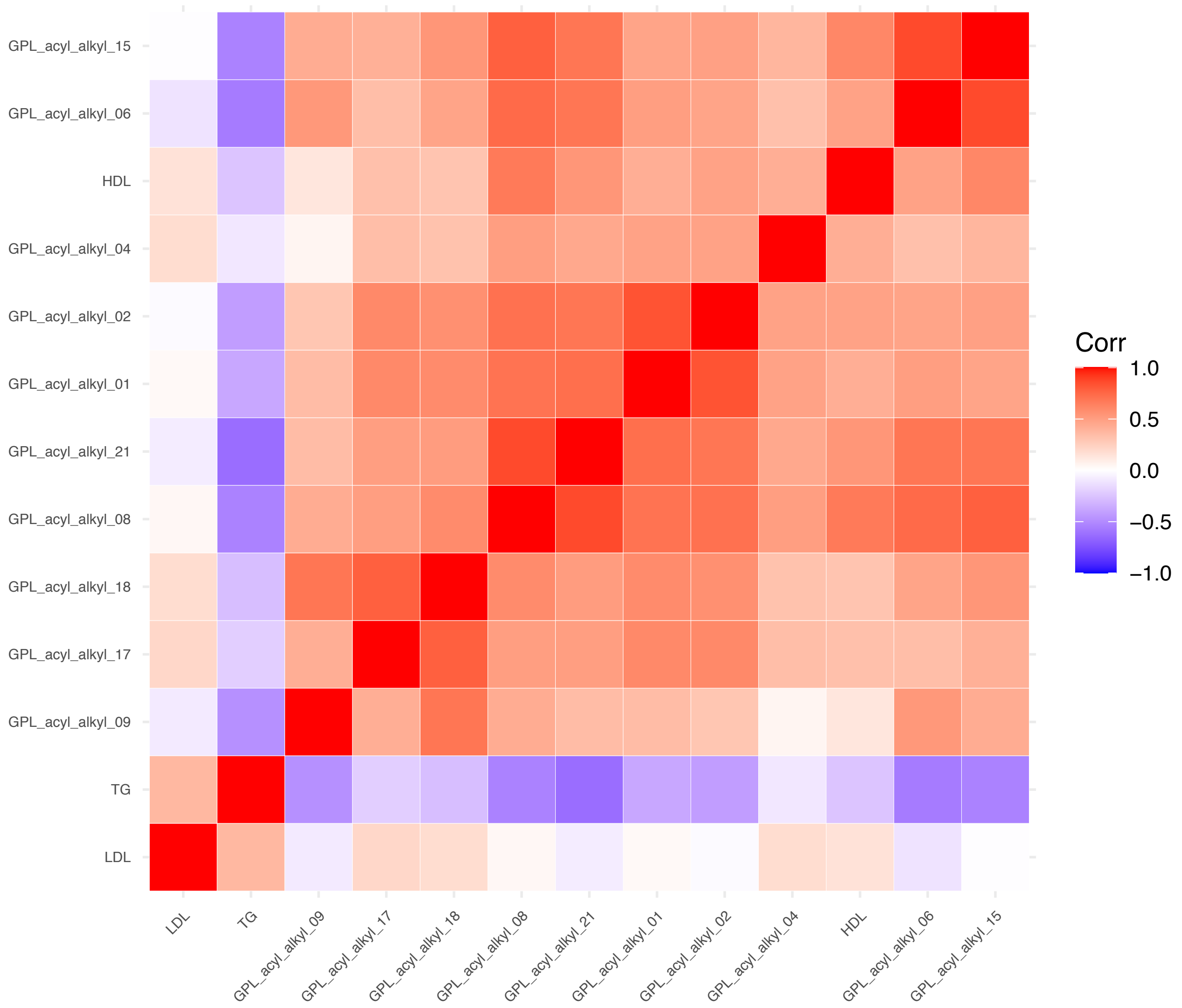

Fig. S6B. Correlation heatmap showing the relationship between associated metabolites in the glycerophospholipid (acyl-acyl) class and traditional measures of metabolic health (Total TAG, LDL and HDL).

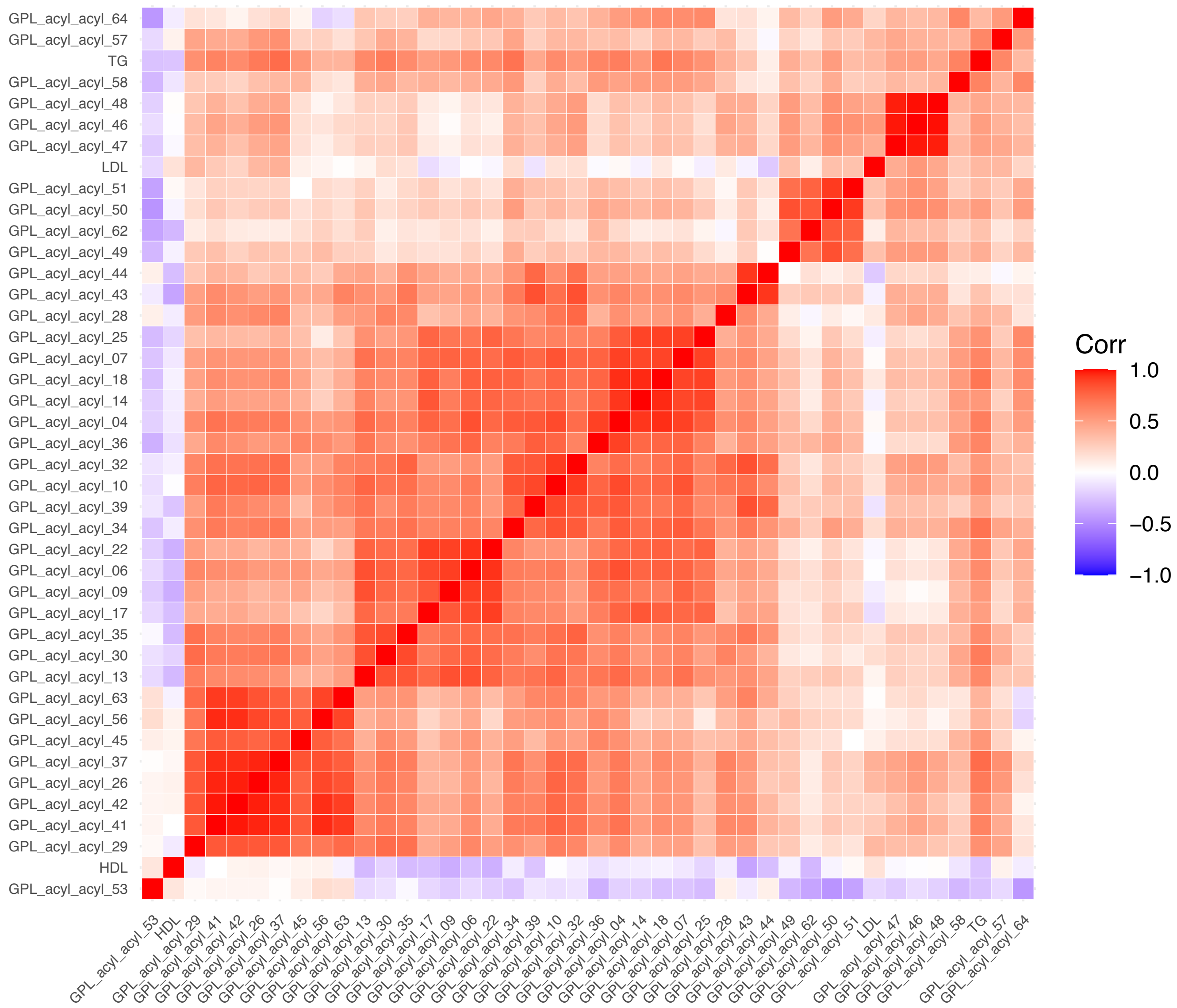

Fig. S7. Principal components analysis score plots. Quality control samples indicated in red. PC = principal component.

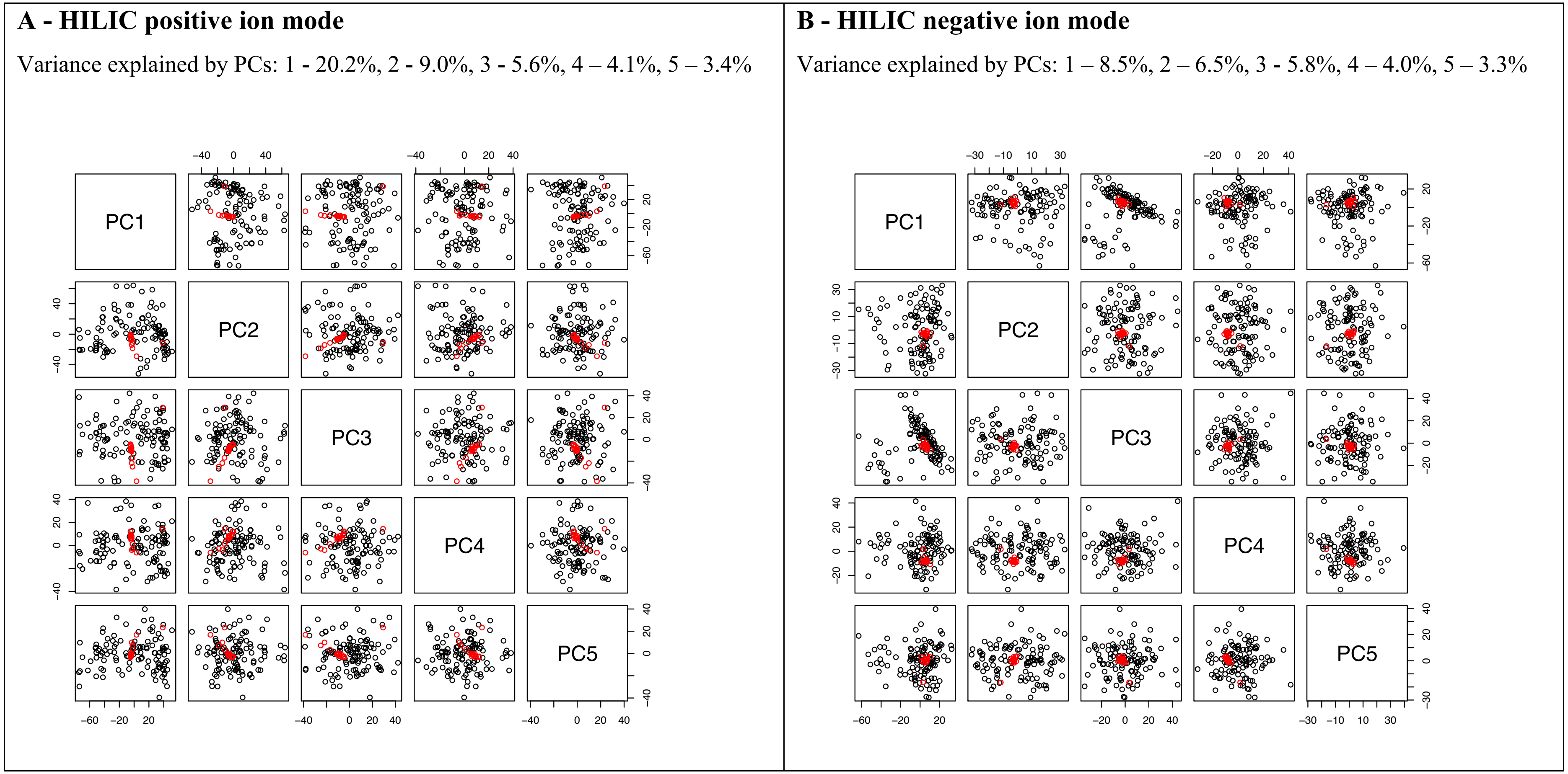

C - Lipids positive ion mode

Variance explained by PCs: 1 – 16.0%, 2 – 7.2%, 3 - 5.9%, 4 – 4.4%, 5 – 3.7%

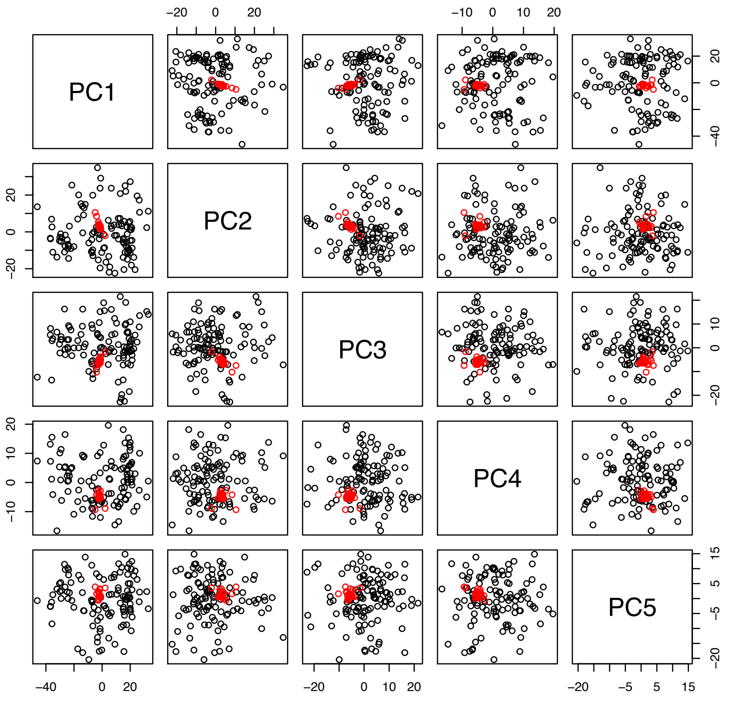

D - Lipids negative ion mode

Variance explained by PCs: 1 – 25.1%, 2 – 10.5%, 3 – 6.0%, 4 – 4.6%, 5 – 4.2%

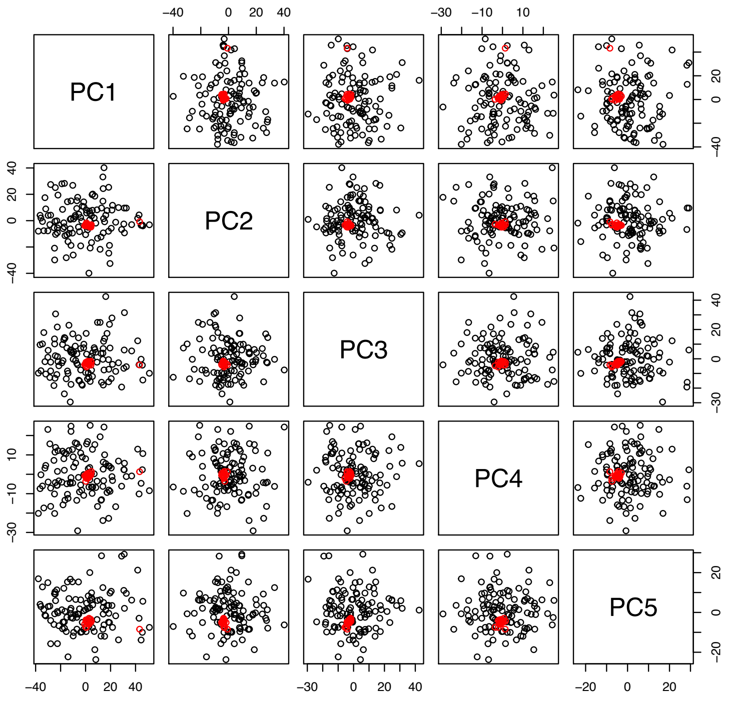

SUPPLEMENTARY TABLES (For Tables S1-S5 see separate Excel file)

Table S6. Results summary by metabolite class

Metabolite Class	Summary of findings (combined dataset)	Relevant biology	Relevance to disease
**TAG**	35 associated with 29 of these showing a decrease in concentration in rs138326449(*APOC3*) carriers. The three TAGs that showed increased levels in carriers of the rare variant were amongst the largest (as determined by molecular mass (*m/z*)).	Range of evidence supporting the role of apoC-III in regulating TAG levels including mouse studies modelling both overexpression of human apoC-III (Ito et al. 1990) and endogenous apoC-III deficiency (Maeda et al. 1994), and the characterization in humans of a range of genetic variants in the *APOC3* gene including our previous NMR study (Drenos et al. 2016).	A series of population-based cohort studies including several meta-analyses have demonstrated that high plasma TAG levels are associated with increased risk of atherosclerotic CVD (Hokanson and Austin 1996; Sarwar et al. 2007). Lipid profiling with respect to cardiovascular events and related phenotypes have shown distinct patterns emerging within TAG subspecies such that the number of carbon atoms and double bonds appear to be relevant. Both a prospective study of CVD (Stegemann et al. 2014) and a randomized dietary intervention trial (Toledo et al. 2017), have shown TAGs to be differentially associated with CVD such that lipids with a lower number of carbon atoms in the acyl chain or with fewer double bonds were associated with a higher risk and those with a higher number of carbon atoms or more double bonds were associated with lower risk. Similar observations have been made with respect to body mass index (Ho et al. 2016) and diabetes prediction (Rhee et al. 2011).
**Acyl-acyl GPL**	39 associated with 38 of these showing a decrease in concentration in the rs138326449(*APOC3*) carriers.	Component of the hydrophilic surface of VLDL. The more common class of GPLs in which the hydrocarbon chain at the *sn-1* position of the glycerol backbone is attached by an ester bond.	Properties depend on the specific molecule but there is some evidence that GPLs are relevant to disease. For example, diacyl-phosphatidylcholines have been associated with obesity (Bagheri et al. 2018, 2019) and mortality (Sigruener et al. 2014a).

Metabolite Class	Summary of findings (combined dataset)	Relevant biology	Relevance to disease
**Acyl-alkyl GPL**	10 associated with all of these showing an increase in concentration in the rs138326449(*APOC3*) carriers.	Component of the hydrophilic surface of VLDL. Peroxisome-derived GPLs that feature an ether bond in place of the ester bond seen in acyl-acyl GPLs and have multiple functions including incorporation into membranes and changes in membrane organisation, fluidity and stability, as endogenous antioxidants and in cell signalling pathways (Dean and Lodhi 2018).	Lower levels of plasmalogens and a specific class of phosphatidylcholines (both acyl-alkyl GPLs) have been associated with a large number of common diseases, including schizophrenia (Huang et al. 2017), Alzheimer's disease (Grimm et al. 2011) obesity and hypertension (Graessler et al. 2009; Pietiläinen et al. 2007), myocardial infarction (Moxon et al. 2017), type 2 diabetes (Razquin et al. 2018), type 1 diabetes development (Orešič et al. 2008) and non-alcoholic fatty liver disease (Orešič et al. 2013). A healthy Nordic diet has been shown to transiently increase the concentrations of antioxidative plasmalogens (Lankinen et al. 2016) and plasmalogen enrichment via batyl alcohol supplementation attenuated atherosclerosis in ApoE- and ApoE/GPx1-deficient mice (Rasmiena et al. 2015).
**DAG**	14 associated with 10 of these showing a decrease in concentration in the rs138326449(*APOC3*) carriers.	DAGs are a metabolic product of TAG lipolysis by lipoprotein lipases. However, DAGs can be synthesised from other sources including de novo synthesis from fatty acids and GPL lysis and DAGs are precursors for GPL synthesis.	Increased concentrations of DAGs have previously been associated with increased risk of CVD (Toledo et al. 2017). DAGs have also been proposed as mediators in the development of insulin resistance associated with obesity and type 2 diabetes, with *APOC3* function highlighted as a potential mechanism for regulating intracellular DAG accumulation in muscle and liver via its inhibition of LPL activity (Erion and Shulman 2010). This hypothesis is in line with the previously documented effect of *APOC3* variants on insulin resistance (Petersen et al. 2010; Waterworth et al. 2003).

Metabolite Class	Summary of findings (combined dataset)	Relevant biology	Relevance to disease
**Ceramide**	24 associated with 11 of these showing a decrease in concentration in the rs138326449(*APOC3*) carriers.	Ceramides are composed of sphingosine and a fatty acid, and are found in high concentrations within the cell membrane of cells (Siskind et al. 2010).	In general, high ceramide levels in biofluids and tissues are associated with lipotoxicity and increased risk of metabolic disorders including diabetes, cardiomyopathy, insulin resistance and atherosclerosis and cardiovascular health (Sigruener et al. 2014b) and with incident major adverse cardiovascular events (Havulinna et al. 2016). Ceramides, especially when used in ratios of two or more, have been shown to be associated with cardiovascular-associated death in two independent studies, independent of other lipid markers and C-reactive protein (Laaksonen et al. 2016). However, there appears to be a dichotomy between various species of ceramides in relation to metabolic phenotypes. Findings of a recent multi-ethnic cohort analysis (N=1,557) suggest that an adverse adiposity and metabolic profile is associated with shorter-chain saturated fatty acid ceramide species whilst a healthier metabolic profile is associated with longer-chain polyunsaturated fatty acid ceramides (Neeland et al. 2018). In particular, the distribution of body fat (visceral versus lower-body subcutaneous) was associated with different subclasses of ceramides (Neeland et al. 2018).

CVD = cardiovascular disease; DAG = diacylglyceride; GPL = glycerophospholipid; NMR = nuclear magnetic resonance; TAG = triacylglyceride; VLDL = very low-density lipoprotein

REFERENCES

Aulchenko, Y. S., Ripke, S., Isaacs, A., & van Duijn, C. M. (2007). GenABEL: an R library for genome-wide association analysis. *Bioinformatics*, *23*(10), 1294–1296. https://doi.org/10.1093/bioinformatics/btm108

Bagheri, M., Djazayery, A., Farzadfar, F., Qi, L., Yekaninejad, M. S., Aslibekyan, S., et al. (2019). Plasma metabolomic profiling of amino acids and polar lipids in Iranian obese adults. *Lipids in Health and Disease*, *18*(1), 94. https://doi.org/10.1186/s12944-019-1037-0

Bagheri, M., Farzadfar, F., Qi, L., Yekaninejad, M. S., Chamari, M., Zeleznik, O. A., et al. (2018). Obesity-Related Metabolomic Profiles and Discrimination of Metabolically Unhealthy Obesity. *Journal of Proteome Research*, *17*(4), 1452–1462. https://doi.org/10.1021/acs.jproteome.7b00802

Benjamini, Y., & Hochberg, Y. (1995). Controlling the False Discovery Rate: A Practical and Powerful Approach to Multiple Testing. *Journal of the Royal Statistical Society. Series B (Methodological)*. WileyRoyal Statistical Society. https://doi.org/10.2307/2346101

Brown, M., Wedge, D. C., Goodacre, R., Kell, D. B., Baker, P. N., Kenny, L. C., et al. (2011). Automated workflows for accurate mass-based putative metabolite identification in LC/MS-derived metabolomic datasets. *Bioinformatics*, *27*(8), 1108–1112. https://doi.org/10.1093/bioinformatics/btr079

Cacciatore, S., Tenori, L., Luchinat, C., Bennett, P. R., & MacIntyre, D. A. (2016). KODAMA: an R package for knowledge discovery and data mining. *Bioinformatics*, *33*(4), btw705. https://doi.org/10.1093/bioinformatics/btw705

Chambers, M. C., Maclean, B., Burke, R., Amodei, D., Ruderman, D. L., Neumann, S., et al. (2012). A cross-platform toolkit for mass spectrometry and proteomics. *Nature Biotechnology*, *30*(10), 918–920. https://doi.org/10.1038/nbt.2377

Chen, H., & Boutros, P. C. (2011). VennDiagram: a package for the generation of highly-customizable Venn and Euler diagrams in R. *BMC Bioinformatics*, *12*(1), 35. https://doi.org/10.1186/1471-2105-12-35

Dean, J. M., & Lodhi, I. J. (2018). Structural and functional roles of ether lipids. *Protein & Cell*, *9*(2), 196–206. https://doi.org/10.1007/s13238-017-0423-5

Drenos, F., Davey Smith, G., Ala-Korpela, M., Kettunen, J., Würtz, P., Soininen, P., et al. (2016). Metabolic Characterization of a Rare Genetic Variation Within *APOC3* and Its Lipoprotein Lipase–Independent EffectsCLINICAL PERSPECTIVE. *Circulation: Cardiovascular Genetics*, *9*(3), 231–239.

https://doi.org/10.1161/CIRCGENETICS.115.001302

Eisen, M. B., Spellman, P. T., Brown, P. O., & Botstein, D. (1998). Cluster analysis and display of genome-wide expression patterns. *Proceedings of the National Academy of Sciences of the United States of America*, *95*(25), 14863–8. https://doi.org/10.1073/pnas.95.25.14863

Erion, D. M., & Shulman, G. I. (2010). Diacylglycerol-mediated insulin resistance. *Nature Medicine*, *16*(4), 400–402. https://doi.org/10.1038/nm0410-400

Frank Dieterle, Alfred Ross, Götz Schlotterbeck, and, & Senn*, H. (2006). Probabilistic Quotient Normalization as Robust Method to Account for Dilution of Complex Biological Mixtures. Application in 1H NMR Metabonomics. https://doi.org/10.1021/AC051632C

Graessler, J., Schwudke, D., Schwarz, P. E. H., Herzog, R., Shevchenko, A., & Bornstein, S. R. (2009). Top-Down Lipidomics Reveals Ether Lipid Deficiency in Blood Plasma of Hypertensive Patients. *PLoS ONE*, *4*(7), e6261. https://doi.org/10.1371/journal.pone.0006261

Grimm, M. O. W., Kuchenbecker, J., Rothhaar, T. L., Grösgen, S., Hundsdörfer, B., Burg, V. K., et al. (2011). Plasmalogen synthesis is regulated via alkyl-dihydroxyacetonephosphate-synthase by amyloid precursor protein processing and is affected in Alzheimer's disease. *Journal of Neurochemistry*, *116*(5), 916–925. https://doi.org/10.1111/j.1471-4159.2010.07070.x

Harris, P. A., Taylor, R., Thielke, R., Payne, J., Gonzalez, N., & Conde, J. G. (2009). Research electronic data capture (REDCap)—A metadata-driven methodology and workflow process for providing translational research informatics support. *Journal of Biomedical Informatics*, *42*(2), 377–381. https://doi.org/10.1016/j.jbi.2008.08.010

Havulinna, A. S., Sysi-Aho, M., Hilvo, M., Kauhanen, D., Hurme, R., Ekroos, K., et al. (2016). Circulating Ceramides Predict Cardiovascular Outcomes in the Population-Based FINRISK 2002 Cohort. *Arteriosclerosis, Thrombosis, and Vascular Biology*, *36*(12), 2424–2430. https://doi.org/10.1161/ATVBAHA.116.307497

Ho, J. E., Larson, M. G., Ghorbani, A., Cheng, S., Chen, M.-H., Keyes, M., et al. (2016). Metabolomic Profiles of Body Mass Index in the Framingham Heart Study Reveal Distinct Cardiometabolic Phenotypes. *PLOS ONE*, *11*(2), e0148361. https://doi.org/10.1371/journal.pone.0148361

Hokanson, J. E., & Austin, M. A. (1996). Plasma triglyceride level is a risk factor for cardiovascular disease independent of high-density lipoprotein cholesterol level: a meta-

analysis of population-based prospective studies. *Journal of cardiovascular risk*, *3*(2), 213–9. http://www.ncbi.nlm.nih.gov/pubmed/8836866. Accessed 12 July 2018

Huang, J. H., Park, H., Iaconelli, J., Berkovitch, S. S., Watmuff, B., McPhie, D., et al. (2017). Unbiased Metabolite Profiling of Schizophrenia Fibroblasts under Stressful Perturbations Reveals Dysregulation of Plasmalogens and Phosphatidylcholines. *Journal of Proteome Research*, *16*(2), 481–493. https://doi.org/10.1021/acs.jproteome.6b00628

Ito, Y., Azrolan, N., O'Connell, A., Walsh, A., & Breslow, J. L. (1990). Hypertriglyceridemia as a result of human apo CIII gene expression in transgenic mice. *Science (New York, N.Y.)*, *249*(4970), 790–3. http://www.ncbi.nlm.nih.gov/pubmed/2167514. Accessed 15 November 2018

Kirwan, J. A., Broadhurst, D. I., Davidson, R. L., & Viant, M. R. (2013). Characterising and correcting batch variation in an automated direct infusion mass spectrometry (DIMS) metabolomics workflow. *Analytical and Bioanalytical Chemistry*, *405*(15), 5147–5157. https://doi.org/10.1007/s00216-013-6856-7

Laaksonen, R., Ekroos, K., Sysi-Aho, M., Hilvo, M., Vihervaara, T., Kauhanen, D., et al. (2016). Plasma ceramides predict cardiovascular death in patients with stable coronary artery disease and acute coronary syndromes beyond LDL-cholesterol. *European Heart Journal*, *37*(25), 1967–1976. https://doi.org/10.1093/eurheartj/ehw148

Lankinen, M., Schwab, U., Kolehmainen, M., Paananen, J., Nygren, H., Seppänen-Laakso, T., et al. (2016). A Healthy Nordic Diet Alters the Plasma Lipidomic Profile in Adults with Features of Metabolic Syndrome in a Multicenter Randomized Dietary Intervention. *The Journal of nutrition*. https://doi.org/10.3945/jn.115.220459

Maeda, N., Li, H., Lee, D., Oliver, P., Quarfordt, S. H., & Osada, J. (1994). Targeted disruption of the apolipoprotein C-III gene in mice results in hypotriglyceridemia and protection from postprandial hypertriglyceridemia. *The Journal of biological chemistry*, *269*(38), 23610–6. http://www.ncbi.nlm.nih.gov/pubmed/8089130. Accessed 15 November 2018

Moxon, J. V., Jones, R. E., Wong, G., Weir, J. M., Mellett, N. A., Kingwell, B. A., et al. (2017). Baseline serum phosphatidylcholine plasmalogen concentrations are inversely associated with incident myocardial infarction in patients with mixed peripheral artery disease presentations. *Atherosclerosis*, *263*, 301–308. https://doi.org/10.1016/j.atherosclerosis.2017.06.925

Murtagh, F., & Legendre, P. (2014). Ward's Hierarchical Agglomerative Clustering Method:

Which Algorithms Implement Ward's Criterion? *Journal of Classification*, *31*, 274–295. https://doi.org/10.1007/s00357-014-9161-z

Neeland, I. J., Singh, S., McGuire, D. K., Vega, G. L., Roddy, T., Reilly, D. F., et al. (2018). Relation of plasma ceramides to visceral adiposity, insulin resistance and the development of type 2 diabetes mellitus: the Dallas Heart Study. *Diabetologia*, *61*(12), 2570–2579. https://doi.org/10.1007/s00125-018-4720-1

Orešič, M., Hyötyläinen, T., Kotronen, A., Gopalacharyulu, P., Nygren, H., Arola, J., et al. (2013). Prediction of non-alcoholic fatty-liver disease and liver fat content by serum molecular lipids. *Diabetologia*, *56*(10), 2266–2274. https://doi.org/10.1007/s00125-013-2981-2

Orešič, M., Simell, S., Sysi-Aho, M., Näntö-Salonen, K., Seppänen-Laakso, T., Parikka, V., et al. (2008). Dysregulation of lipid and amino acid metabolism precedes islet autoimmunity in children who later progress to type 1 diabetes. *The Journal of Experimental Medicine*, *205*(13), 2975–2984. https://doi.org/10.1084/jem.20081800

Peake, D., Kiyonami, R., Yokoi, Y., Fukamachi, Y., & Huang, Y. (2015). Processing of a Complex Lipid Dataset for the NIST Inter-laboratory Comparison Exercise for Lipidomics Measurements in Human Serum and Plasma. In *LIPID MAPS Annual Meeting*. https://www.thermofisher.com/blog/proteomics/processing-a-complex-lipid-data-set-with-lipidsearch-software/

Petersen, K. F., Dufour, S., Hariri, A., Nelson-Williams, C., Foo, J. N., Zhang, X.-M., et al. (2010). Apolipoprotein C3 Gene Variants in Nonalcoholic Fatty Liver Disease. *New England Journal of Medicine*, *362*(12), 1082–1089. https://doi.org/10.1056/NEJMoa0907295

Pietiläinen, K. H., Sysi-Aho, M., Rissanen, A., Seppänen-Laakso, T., Yki-Järvinen, H., Kaprio, J., & Orešič, M. (2007). Acquired Obesity Is Associated with Changes in the Serum Lipidomic Profile Independent of Genetic Effects – A Monozygotic Twin Study. *PLoS ONE*, *2*(2), e218. https://doi.org/10.1371/journal.pone.0000218

R Core Team. (2019). R: A Language and Environment for Statistical Computing. Vienna, Austria. https://www.r-project.org

Rasmiena, A. A., Barlow, C. K., Stefanovic, N., Huynh, K., Tan, R., Sharma, A., et al. (2015). Plasmalogen modulation attenuates atherosclerosis in ApoE- and ApoE/GPx1-deficient mice. *Atherosclerosis*, *243*(2), 598–608. https://doi.org/10.1016/j.atherosclerosis.2015.10.096

Razquin, C., Toledo, E., Clish, C. B., Ruiz-Canela, M., Dennis, C., Corella, D., et al. (2018).

Plasma Lipidomic Profiling and Risk of Type 2 Diabetes in the PREDIMED Trial. *Diabetes Care*, dc180840. https://doi.org/10.2337/dc18-0840

Rhee, E. P., Cheng, S., Larson, M. G., Walford, G. A., Lewis, G. D., McCabe, E., et al. (2011). Lipid profiling identifies a triacylglycerol signature of insulin resistance and improves diabetes prediction in humans. *Journal of Clinical Investigation*, *121*(4), 1402–1411. https://doi.org/10.1172/JCI44442

Royston, J. P. (1982a). Algorithm AS 181: The W Test for Normality. *Applied Statistics*, *31*(2), 176–180. http://sci2s.ugr.es/keel/pdf/algorithm/articulo/royston1982.pdf. Accessed 12 April 2018

Royston, J. P. (1982b). An Extension of Shapiro and Wilk's W Test for Normality to Large Samples. *Journal of the Royal Statistical Society. Series C (Applied Statistics)*, *31*(2), 115–124. http://www.jstor.org/stable/2347973

Royston, P. (1995). Remark AS R94: A Remark on Algorithm AS 181: The W-test for Normality. *Applied Statistics*, *44*(4), 547. https://doi.org/10.2307/2986146

Sarwar, N., Danesh, J., Eiriksdottir, G., Sigurdsson, G., Wareham, N., Bingham, S., et al. (2007). Triglycerides and the Risk of Coronary Heart Disease: 10 158 Incident Cases Among 262 525 Participants in 29 Western Prospective Studies. *Circulation*, *115*(4), 450–458. https://doi.org/10.1161/CIRCULATIONAHA.106.637793

Sedlmeier, A., Kluttig, A., Giegling, I., Prehn, C., Adamski, J., Kastenmüller, G., & Lacruz, M. E. (2018). The human metabolic profile reflects macro- and micronutrient intake distinctly according to fasting time. *Scientific Reports*, *8*(1), 12262. https://doi.org/10.1038/s41598-018-30764-4

Sigruener, A., Kleber, M. E., Heimerl, S., Liebisch, G., Schmitz, G., & Maerz, W. (2014a). Glycerophospholipid and Sphingolipid Species and Mortality: The Ludwigshafen Risk and Cardiovascular Health (LURIC) Study. *PLoS ONE*, *9*(1), e85724. https://doi.org/10.1371/journal.pone.0085724

Sigruener, A., Kleber, M. E., Heimerl, S., Liebisch, G., Schmitz, G., & Maerz, W. (2014b). Glycerophospholipid and Sphingolipid Species and Mortality: The Ludwigshafen Risk and Cardiovascular Health (LURIC) Study. *PLoS ONE*, *9*(1), e85724. https://doi.org/10.1371/journal.pone.0085724

Siskind, L. J., Mullen, T. D., & Obeid, L. M. (2010). The Role of Ceramide in Cell Regulation. *Handbook of Cell Signaling*, 1201–1211. https://doi.org/10.1016/B978-0-12-374145-5.00148-0

Smith, C. A., Want, E. J., O'Maille, G., Abagyan, R., & Siuzdak, G. (2006).

XCMS: Processing Mass Spectrometry Data for Metabolite Profiling Using Nonlinear Peak Alignment, Matching, and Identification. *Analytical Chemistry*, *78*(3), 779–787. https://doi.org/10.1021/ac051437y

Stegemann, C., Pechlaner, R., Willeit, P., Langley, S. R., Mangino, M., Mayr, U., et al. (2014). Lipidomics Profiling and Risk of Cardiovascular Disease in the Prospective Population-Based Bruneck Study. *Circulation*, *129*(18), 1821–1831. https://doi.org/10.1161/CIRCULATIONAHA.113.002500

Stekhoven, D. J., & Buhlmann, P. (2012). MissForest--non-parametric missing value imputation for mixed-type data. *Bioinformatics*, *28*(1), 112–118. https://doi.org/10.1093/bioinformatics/btr597

Sumner, L. W., Amberg, A., Barrett, D., Beale, M. H., Beger, R., Daykin, C. A., et al. (2007). Proposed minimum reporting standards for chemical analysis. *Metabolomics*, *3*(3), 211–221. https://doi.org/10.1007/s11306-007-0082-2

Toledo, E., Wang, D. D., Ruiz-Canela, M., Clish, C. B., Razquin, C., Zheng, Y., et al. (2017). Plasma lipidomic profiles and cardiovascular events in a randomized intervention trial with the Mediterranean diet. *The American Journal of Clinical Nutrition*, *106*(4), 973–983. https://doi.org/10.3945/ajcn.116.151159

Vazquez, A. I., Bates, D. M., Rosa, G. J. M., Gianola, D., & Weigel, K. A. (2010). Technical note: An R package for fitting generalized linear mixed models in animal breeding1. *Journal of Animal Science*, *88*(2), 497–504. https://doi.org/10.2527/jas.2009-1952

Ward, J. H. (1963). Hierarchical Grouping to Optimize an Objective Function. *Journal of the American Statistical Association*, *58*(301), 236–244. https://doi.org/10.1080/01621459.1963.10500845

Waterworth, D. M., Talmud, P. J., Luan, J., Flavell, D. M., Byrne, C. D., Humphries, S. E., & Wareham, N. J. (2003). Variants in the APOC3 promoter insulin responsive element modulate insulin secretion and lipids in middle-aged men. *Biochimica et biophysica acta*, *1637*(3), 200–6. http://www.ncbi.nlm.nih.gov/pubmed/12697301. Accessed 23 January 2019

Wickham, H. (2016). *ggplot2 : Elegant Graphics for Data Analysis* (2nd ed.). New York: Springer International Publishing. http://ggplot2.org. Accessed 7 August 2018

Zhao, S., Guo, Y., Sheng, Q., & Shyr, Y. (2014). Heatmap3: an improved heatmap package with more powerful and convenient features. *BMC Bioinformatics*, *15*(Suppl 10), P16. https://doi.org/10.1186/1471-2105-15-S10-P16
