# Supplementary material for: Metabolic characterisation of disturbances in the APOC3/triglyceride-rich lipoprotein pathway through sample-based recall by genotype
Source: Metabolomics. 2020 Jun 3;16(6):69. doi: 10.1007/s11306-020-01689-9 (PMC7270992; doi:10.1007/s11306-020-01689-9)
Supplement: Supplementary file 2 — Supplementary file2 (PDF 74 kb) [file 11306_2020_1689_MOESM2_ESM.pdf]

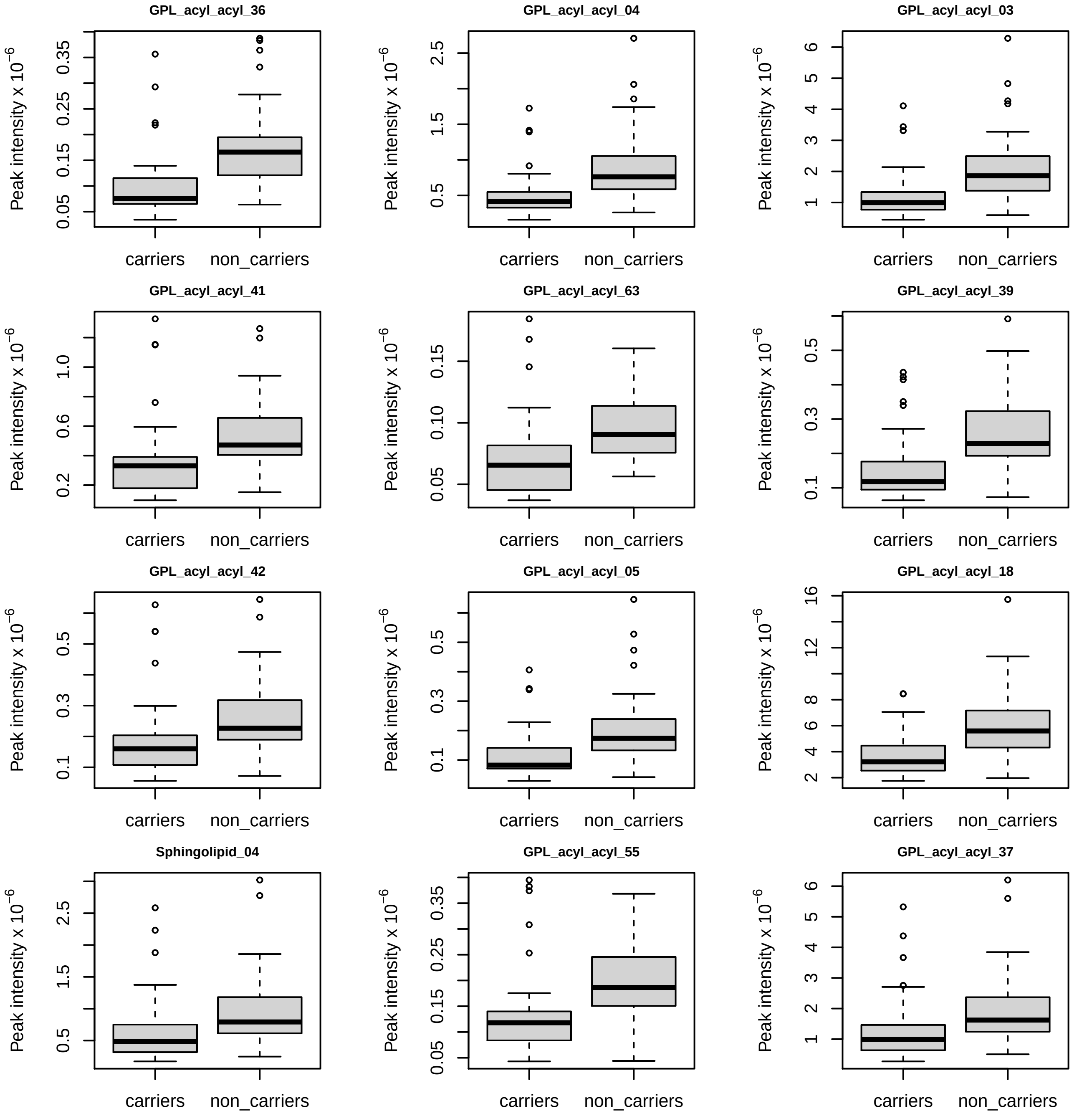
GPL_acyl_acyl_36
Peak intensity x 10^{-6}
0.05 0.15 0.25 0.35
carriers
non_carriers
GPL_acyl_acyl_04
Peak intensity x 10^{-6}
0.5 1.5 2.5
carriers
non_carriers
GPL_acyl_acyl_03
Peak intensity x 10^{-6}
1 2 3 4 5 6
carriers
non_carriers
GPL_acyl_acyl_41
Peak intensity x 10^{-6}
0.2 0.6 1.0
carriers
non_carriers
GPL_acyl_acyl_63
Peak intensity x 10^{-6}
0.05 0.10 0.15
carriers
non_carriers
GPL_acyl_acyl_39
Peak intensity x 10^{-6}
0.1 0.3 0.5
carriers
non_carriers
GPL_acyl_acyl_42
Peak intensity x 10^{-6}
0.1 0.3 0.5
carriers
non_carriers
GPL_acyl_acyl_05
Peak intensity x 10^{-6}
0.1 0.3 0.5
carriers
non_carriers
GPL_acyl_acyl_18
Peak intensity x 10^{-6}
2 4 6 8 12 16
carriers
non_carriers
Sphingolipid_04
Peak intensity x 10^{-6}
0.5 1.5 2.5
carriers
non_carriers
GPL_acyl_acyl_55
Peak intensity x 10^{-6}
0.05 0.15 0.25 0.35
carriers
non_carriers
GPL_acyl_acyl_37
Peak intensity x 10^{-6}
1 2 3 4 5 6
carriers
non_carriers

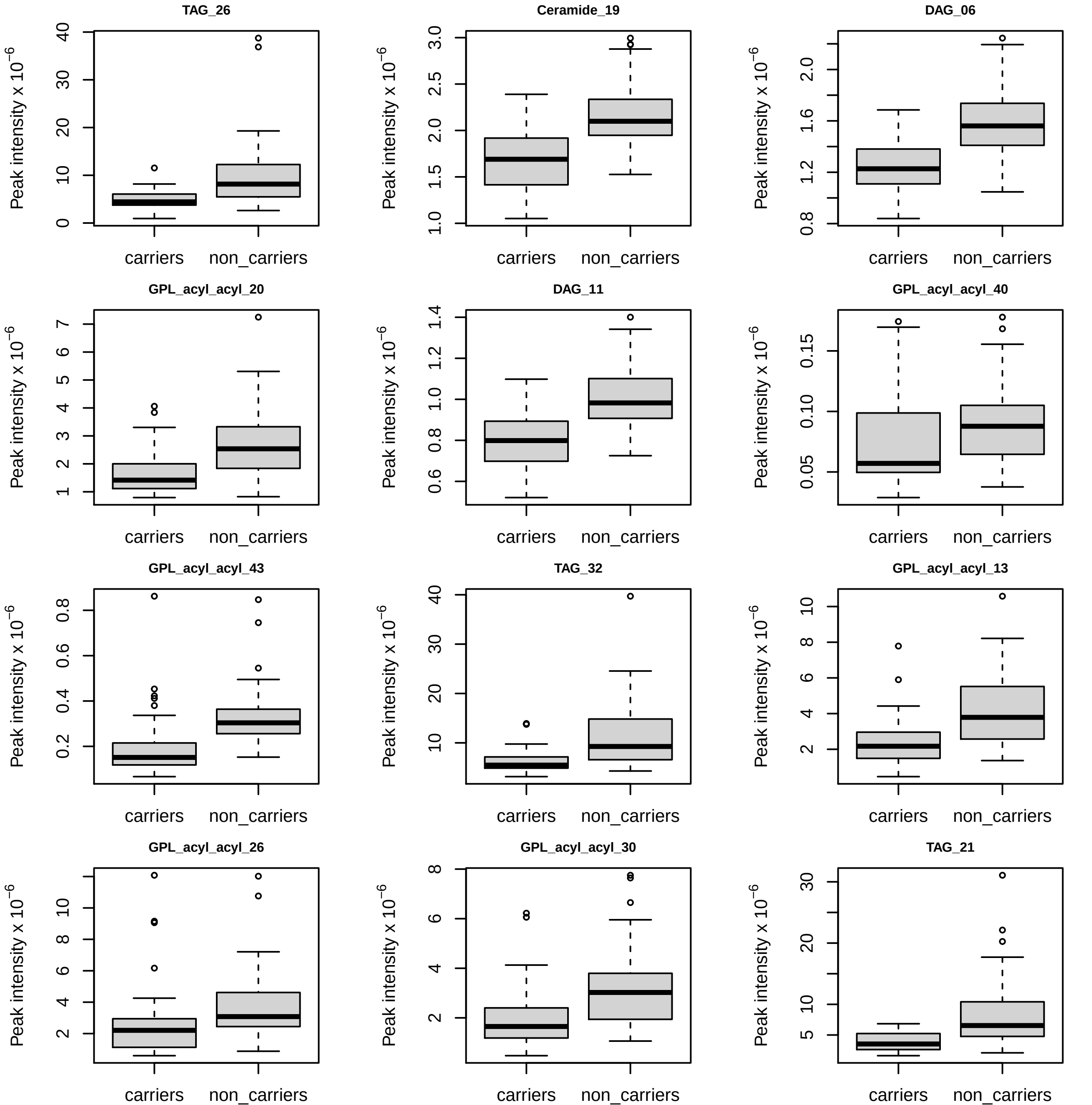

TAG_26
Ceramide_19
DAG_06
GPL_acyl_acyl_20
DAG_11
GPL_acyl_acyl_40
GPL_acyl_acyl_43
TAG_32
GPL_acyl_acyl_13
GPL_acyl_acyl_26
GPL_acyl_acyl_30
TAG_21
Peak intensity x 10^{-6}
carriers
non_carriers

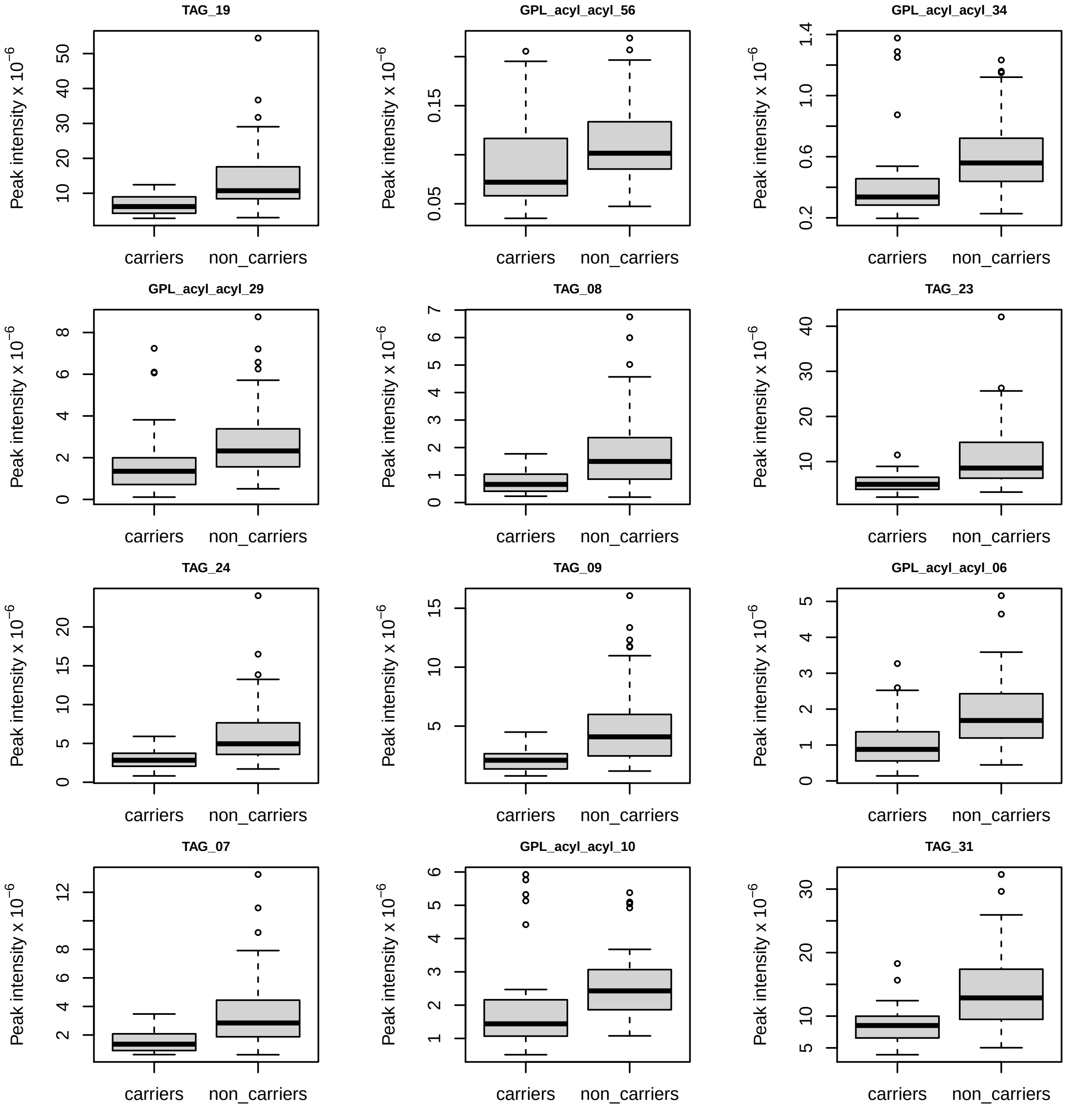
TAG_19
GPL_acyl_acyl_56
GPL_acyl_acyl_34
GPL_acyl_acyl_29
TAG_08
TAG_23
TAG_24
TAG_09
GPL_acyl_acyl_06
TAG_07
GPL_acyl_acyl_10
TAG_31
Peak intensity x 10^{-6}
carriers
non_carriers

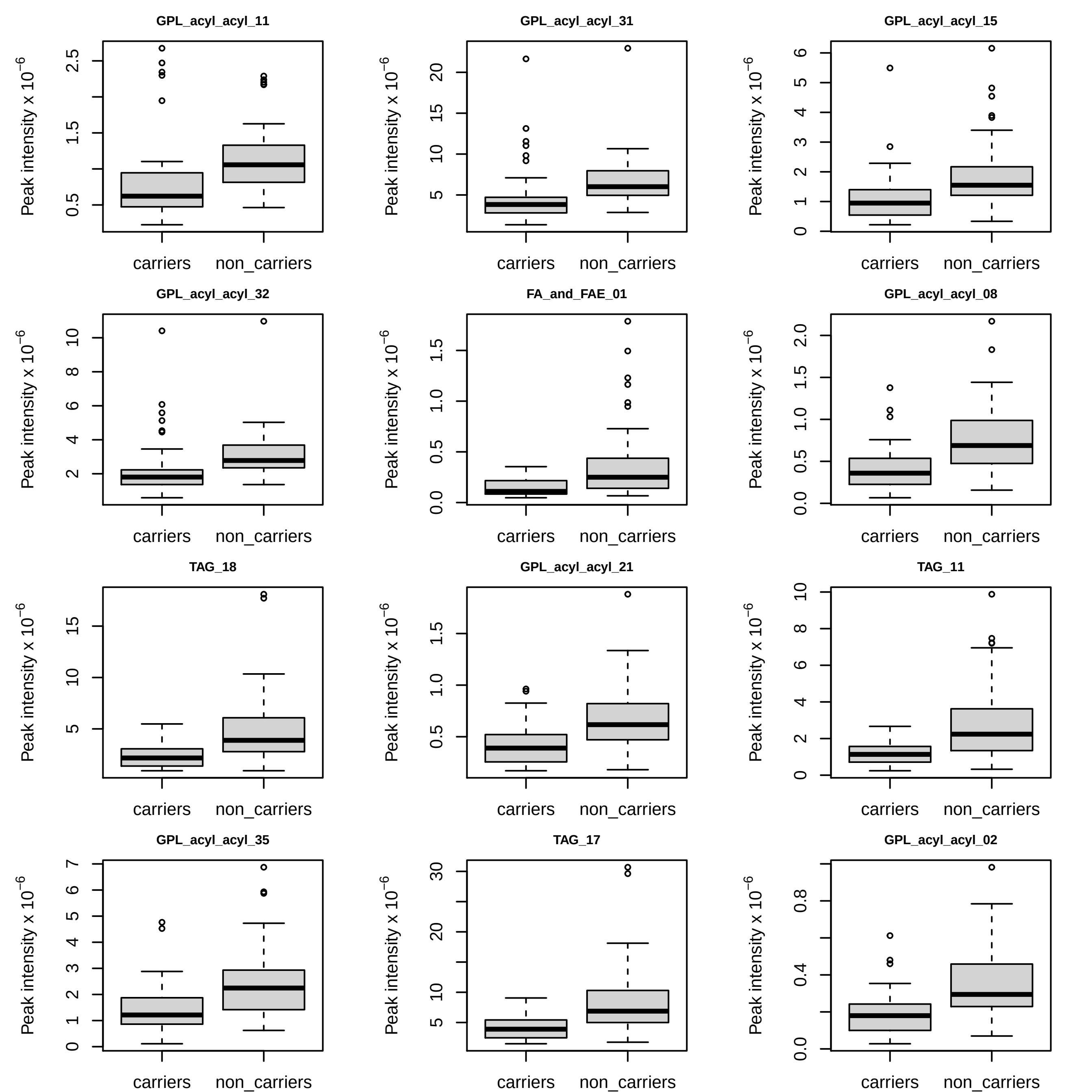

GPL_acyl_acyl_11
GPL_acyl_acyl_31
GPL_acyl_acyl_15
GPL_acyl_acyl_32
FA_and_FAE_01
GPL_acyl_acyl_08
TAG_18
GPL_acyl_acyl_21
TAG_11
GPL_acyl_acyl_35
TAG_17
GPL_acyl_acyl_02
Peak intensity x 10^{-6}
carriers
non_carriers

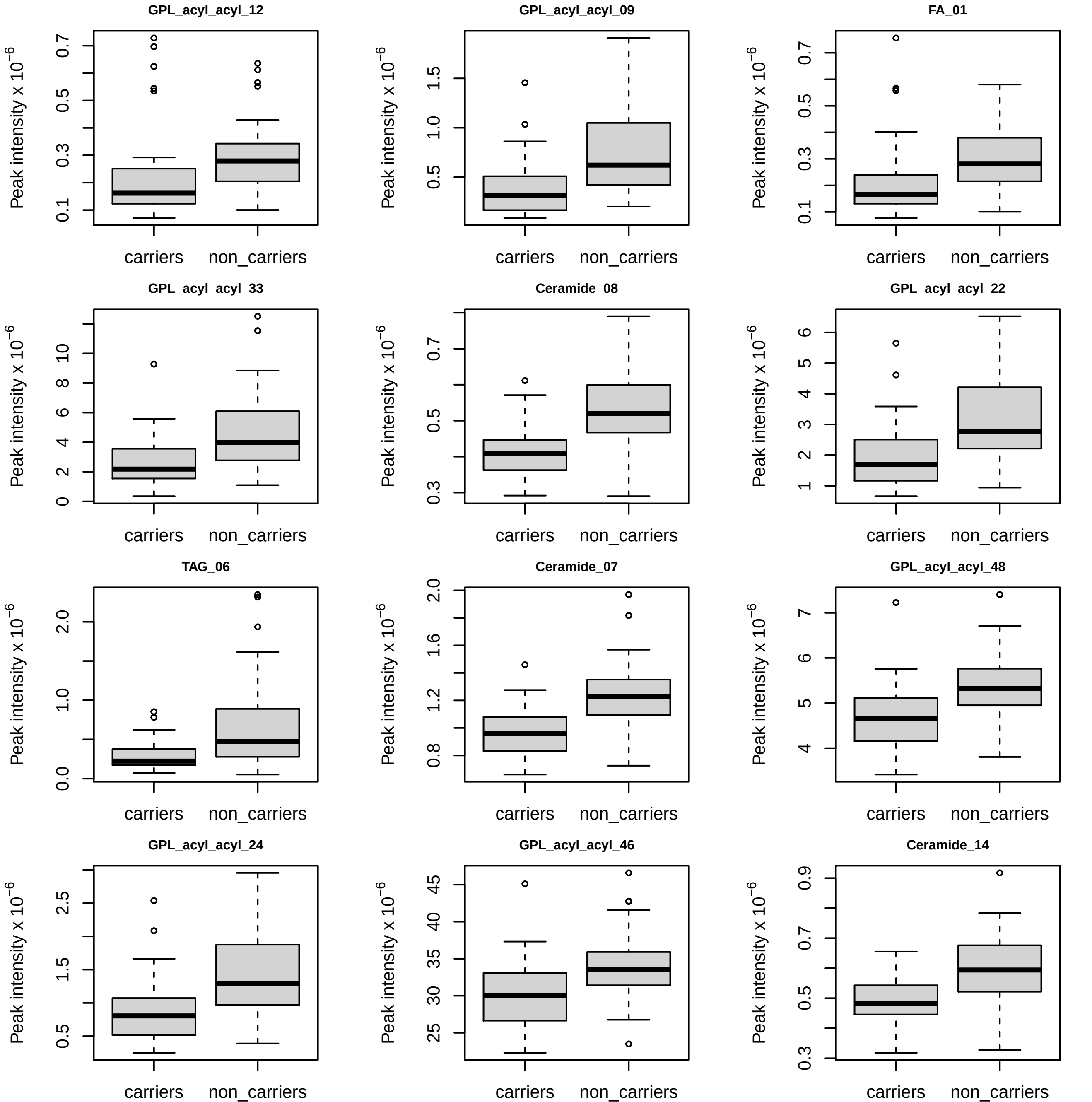
GPL_acyl_acyl_12
GPL_acyl_acyl_09
FA_01
GPL_acyl_acyl_33
Ceramide_08
GPL_acyl_acyl_22
TAG_06
Ceramide_07
GPL_acyl_acyl_48
GPL_acyl_acyl_24
GPL_acyl_acyl_46
Ceramide_14
Peak intensity x 10^{-6}
carriers
non_carriers

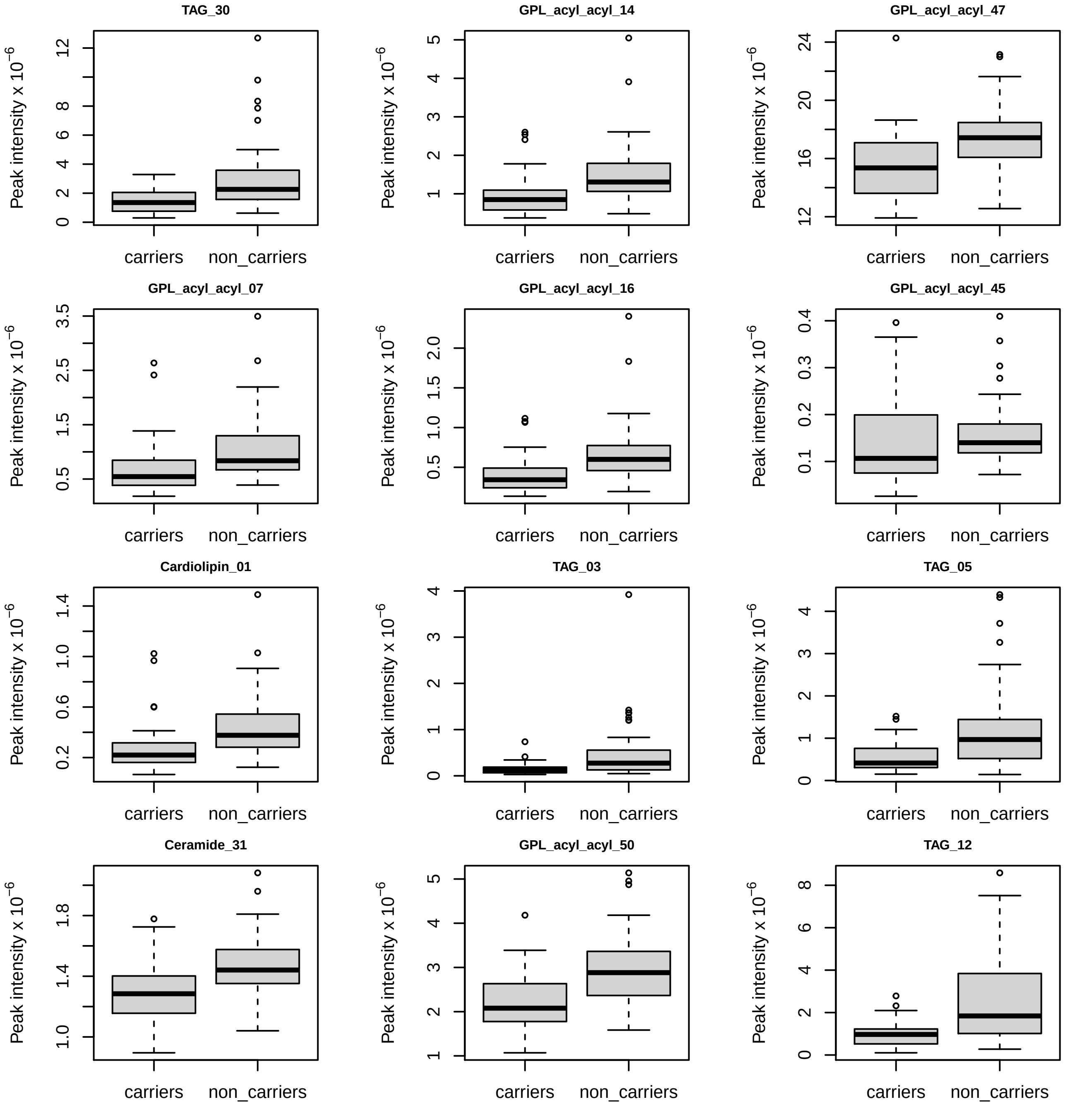

TAG_30
GPL_acyl_acyl_14
GPL_acyl_acyl_47
GPL_acyl_acyl_07
GPL_acyl_acyl_16
GPL_acyl_acyl_45
Cardiolipin_01
TAG_03
TAG_05
Ceramide_31
GPL_acyl_acyl_50
TAG_12
Peak intensity x 10^{-6}
carriers
non_carriers

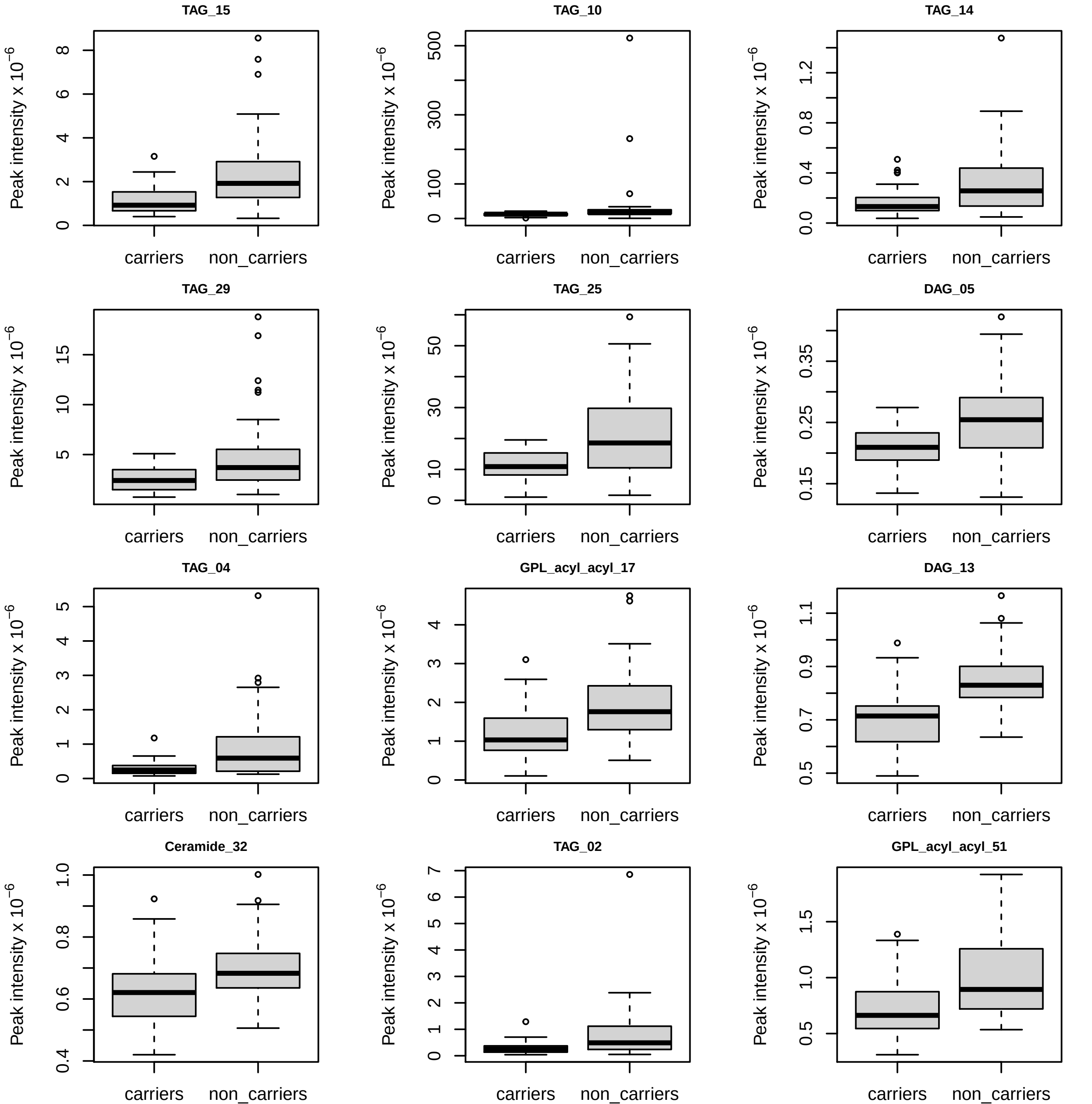

TAG_15
TAG_10
TAG_14
TAG_29
TAG_25
DAG_05
TAG_04
GPL_acyl_acyl_17
DAG_13
Ceramide_32
TAG_02
GPL_acyl_acyl_51
Peak intensity x 10^{-6}
carriers
non_carriers

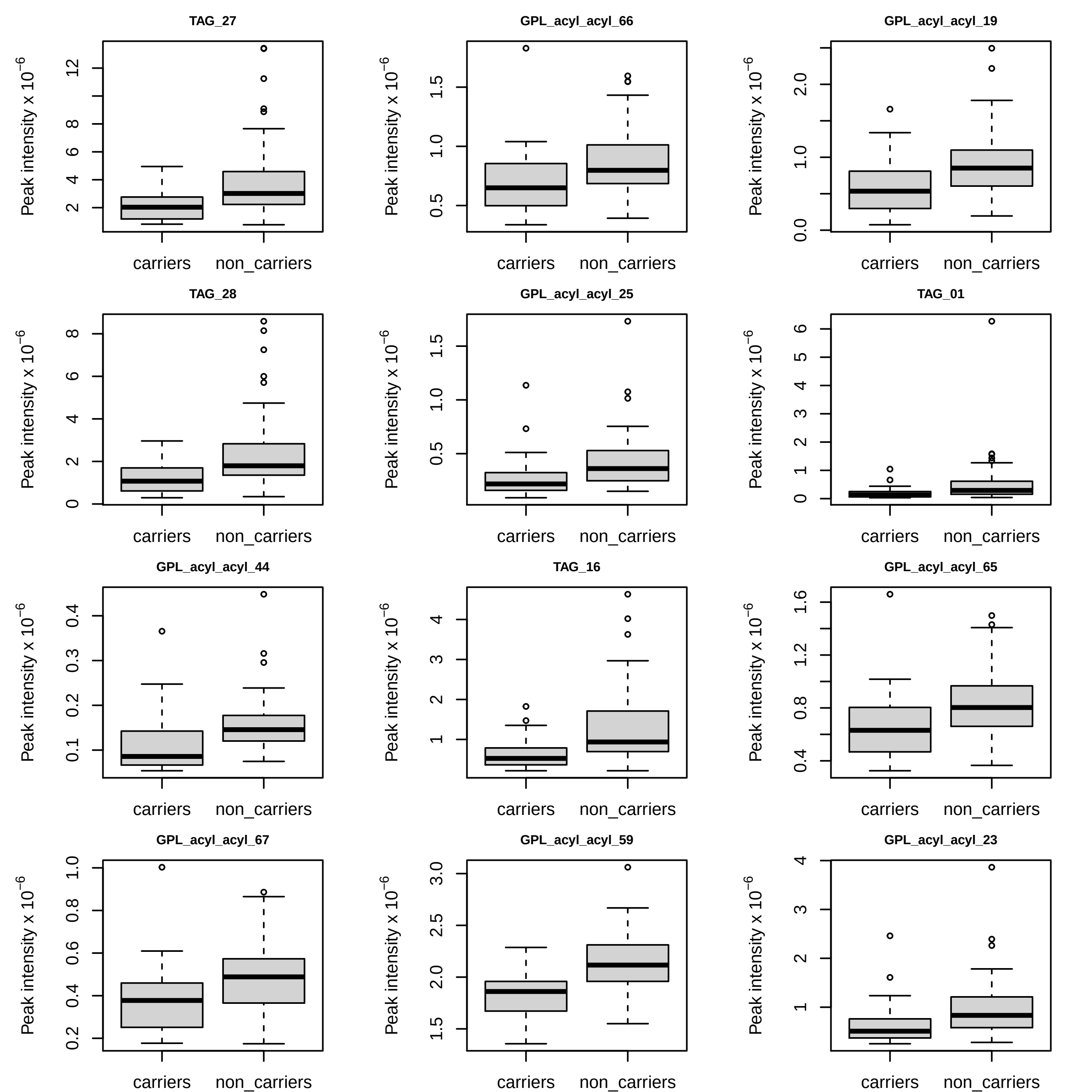
TAG_27
GPL_acyl_acyl_66
GPL_acyl_acyl_19
TAG_28
GPL_acyl_acyl_25
TAG_01
GPL_acyl_acyl_44
TAG_16
GPL_acyl_acyl_65
GPL_acyl_acyl_67
GPL_acyl_acyl_59
GPL_acyl_acyl_23
Peak intensity x 10^{-6}
carriers
non_carriers

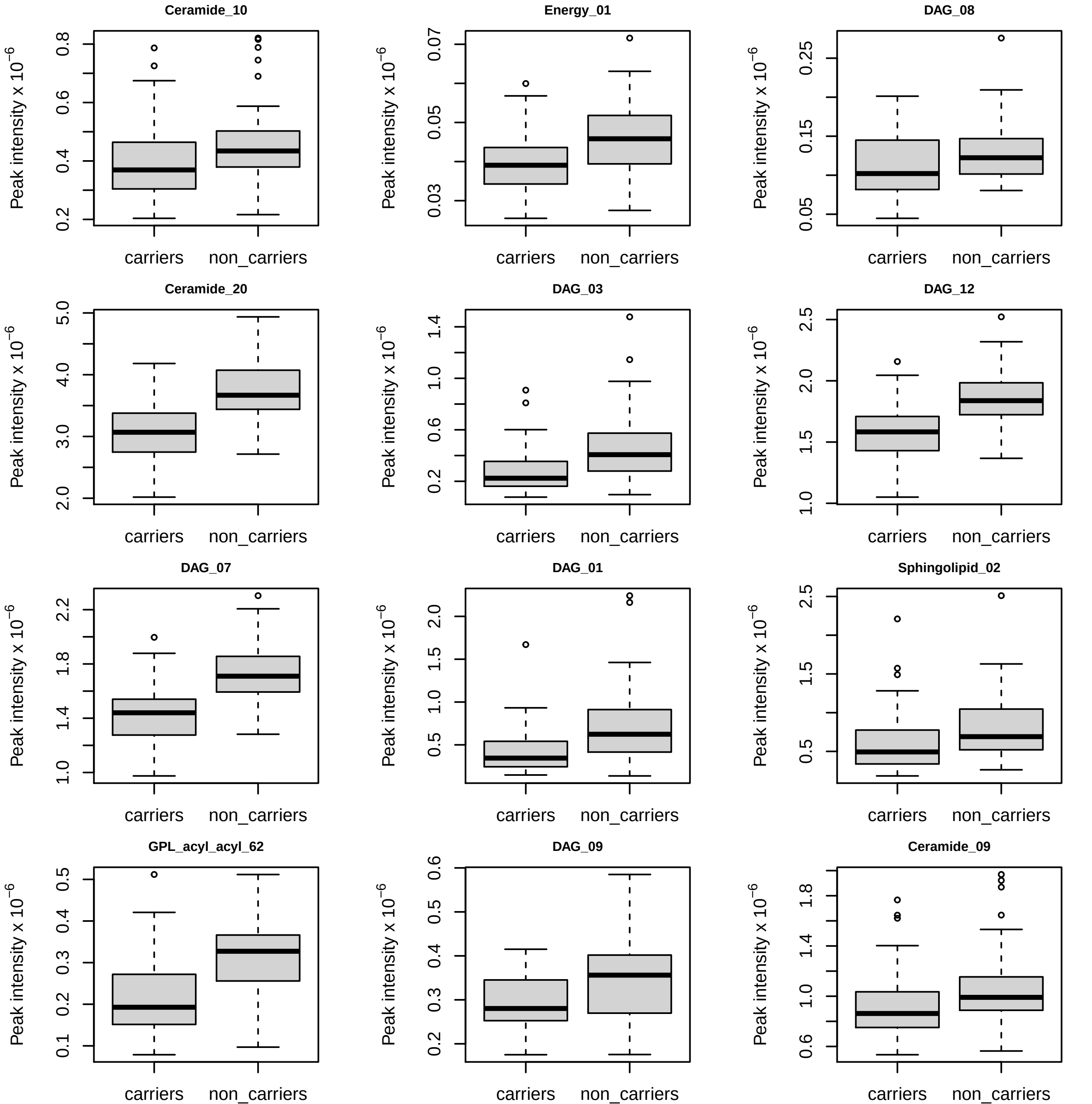
Ceramide_10
Peak intensity x 10^{-6}
0.2 0.4 0.6 0.8
carriers non_carriers
Energy_01
Peak intensity x 10^{-6}
0.03 0.05 0.07
carriers non_carriers
DAG_08
Peak intensity x 10^{-6}
0.05 0.15 0.25
carriers non_carriers
Ceramide_20
Peak intensity x 10^{-6}
2.0 3.0 4.0 5.0
carriers non_carriers
DAG_03
Peak intensity x 10^{-6}
0.2 0.6 1.0 1.4
carriers non_carriers
DAG_12
Peak intensity x 10^{-6}
1.0 1.5 2.0 2.5
carriers non_carriers
DAG_07
Peak intensity x 10^{-6}
1.0 1.4 1.8 2.2
carriers non_carriers
DAG_01
Peak intensity x 10^{-6}
0.5 1.0 1.5 2.0
carriers non_carriers
Sphingolipid_02
Peak intensity x 10^{-6}
0.5 1.5 2.5
carriers non_carriers
GPL_acyl_acyl_62
Peak intensity x 10^{-6}
0.1 0.2 0.3 0.4 0.5
carriers non_carriers
DAG_09
Peak intensity x 10^{-6}
0.2 0.3 0.4 0.5 0.6
carriers non_carriers
Ceramide_09
Peak intensity x 10^{-6}
0.6 1.0 1.4 1.8
carriers non_carriers

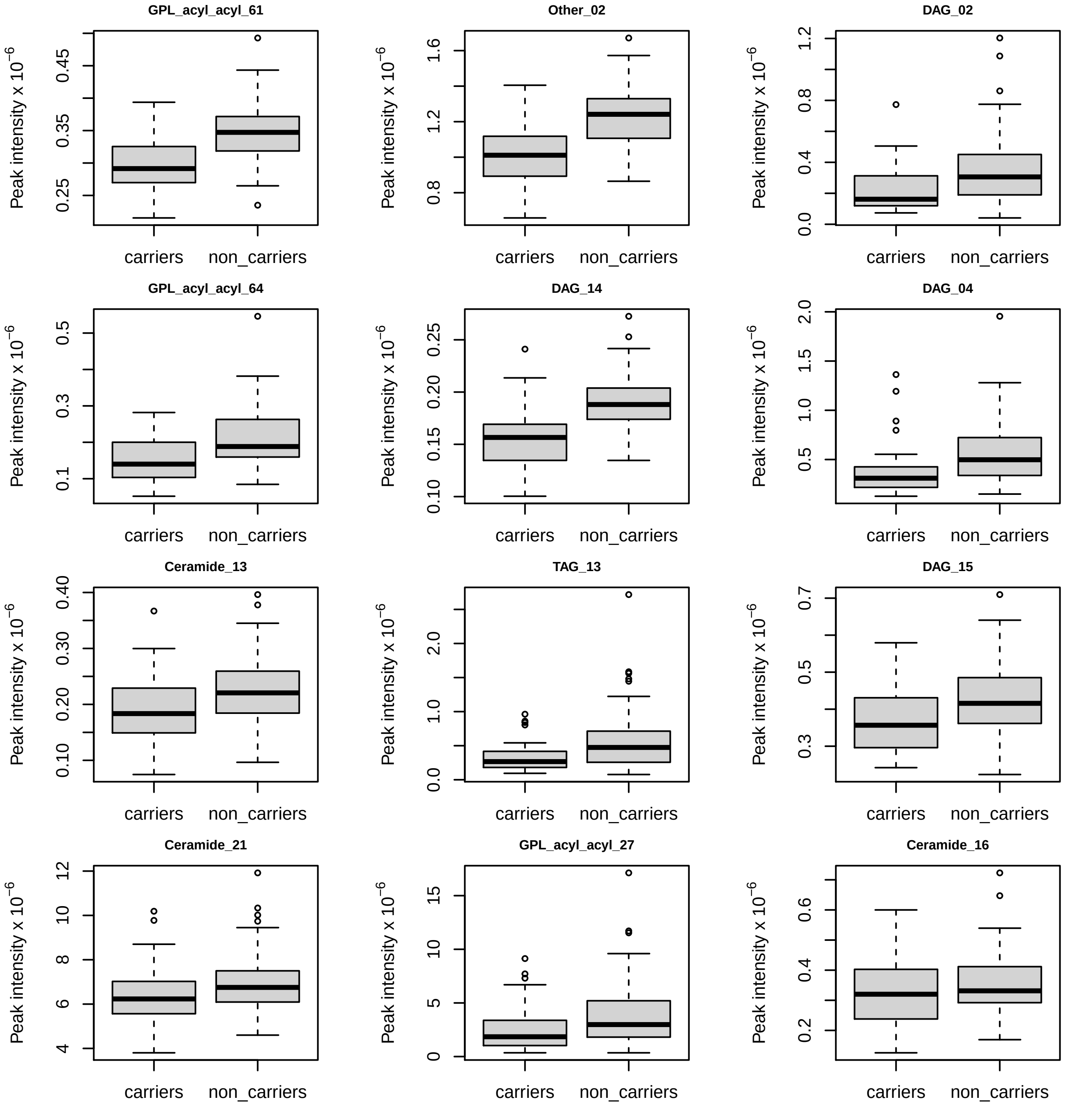
GPL_acyl_acyl_61
Peak intensity x 10^{-6}
0.25
0.35
0.45
carriers
non_carriers
Other_02
Peak intensity x 10^{-6}
0.8
1.2
1.6
carriers
non_carriers
DAG_02
Peak intensity x 10^{-6}
0.0
0.4
0.8
1.2
carriers
non_carriers
GPL_acyl_acyl_64
Peak intensity x 10^{-6}
0.1
0.3
0.5
carriers
non_carriers
DAG_14
Peak intensity x 10^{-6}
0.10
0.15
0.20
0.25
carriers
non_carriers
DAG_04
Peak intensity x 10^{-6}
0.5
1.0
1.5
2.0
carriers
non_carriers
Ceramide_13
Peak intensity x 10^{-6}
0.10
0.20
0.30
0.40
carriers
non_carriers
TAG_13
Peak intensity x 10^{-6}
0.0
1.0
2.0
carriers
non_carriers
DAG_15
Peak intensity x 10^{-6}
0.3
0.5
0.7
carriers
non_carriers
Ceramide_21
Peak intensity x 10^{-6}
4
6
8
10
12
carriers
non_carriers
GPL_acyl_acyl_27
Peak intensity x 10^{-6}
0
5
10
15
carriers
non_carriers
Ceramide_16
Peak intensity x 10^{-6}
0.2
0.4
0.6
carriers
non_carriers

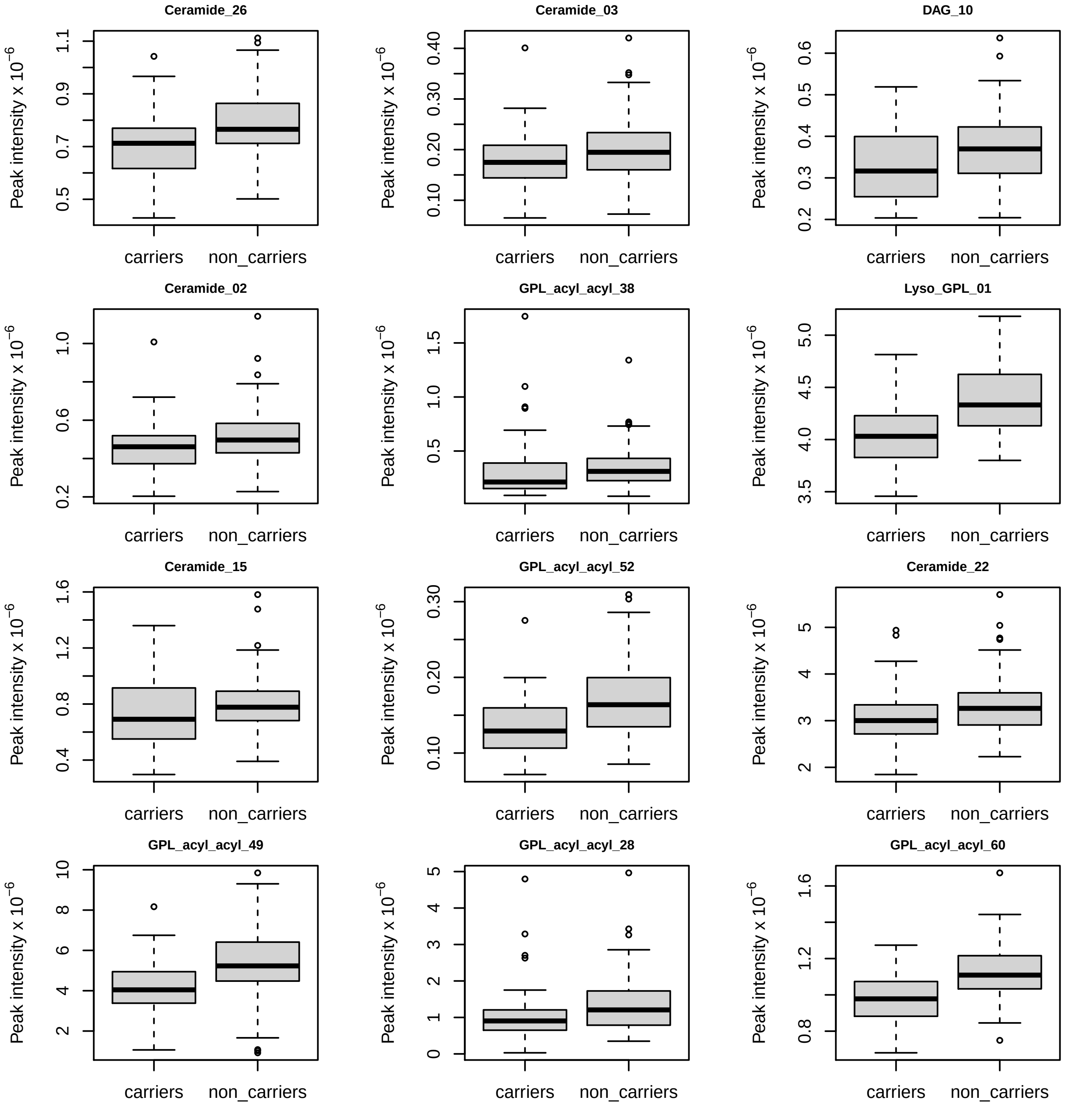

Ceramide_26
Peak intensity x 10^{-6}
0.5 0.7 0.9 1.1
carriers non_carriers
Ceramide_03
Peak intensity x 10^{-6}
0.10 0.20 0.30 0.40
carriers non_carriers
DAG_10
Peak intensity x 10^{-6}
0.2 0.3 0.4 0.5 0.6
carriers non_carriers
Ceramide_02
Peak intensity x 10^{-6}
0.2 0.6 1.0
carriers non_carriers
GPL_acyl_acyl_38
Peak intensity x 10^{-6}
0.5 1.0 1.5
carriers non_carriers
Lyso_GPL_01
Peak intensity x 10^{-6}
3.5 4.0 4.5 5.0
carriers non_carriers
Ceramide_15
Peak intensity x 10^{-6}
0.4 0.8 1.2 1.6
carriers non_carriers
GPL_acyl_acyl_52
Peak intensity x 10^{-6}
0.10 0.20 0.30
carriers non_carriers
Ceramide_22
Peak intensity x 10^{-6}
2 3 4 5
carriers non_carriers
GPL_acyl_acyl_49
Peak intensity x 10^{-6}
2 4 6 8 10
carriers non_carriers
GPL_acyl_acyl_28
Peak intensity x 10^{-6}
0 1 2 3 4 5
carriers non_carriers
GPL_acyl_acyl_60
Peak intensity x 10^{-6}
0.8 1.2 1.6
carriers non_carriers

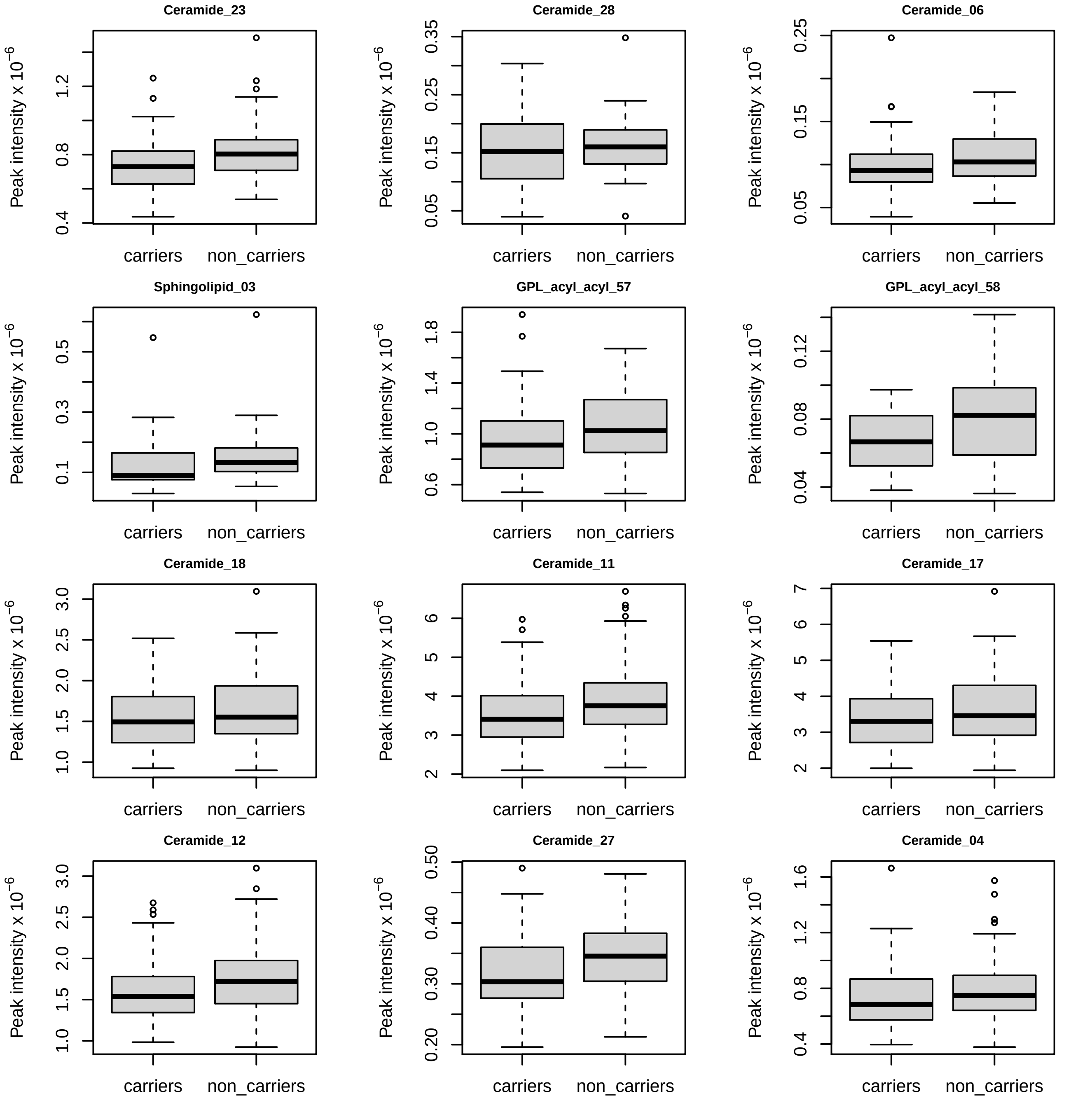

Ceramide_23
Peak intensity x 10^{-6}
0.4
0.8
1.2
carriers
non_carriers
Ceramide_28
Peak intensity x 10^{-6}
0.05
0.15
0.25
0.35
carriers
non_carriers
Ceramide_06
Peak intensity x 10^{-6}
0.05
0.15
0.25
carriers
non_carriers
Sphingolipid_03
Peak intensity x 10^{-6}
0.1
0.3
0.5
carriers
non_carriers
GPL_acyl_acyl_57
Peak intensity x 10^{-6}
0.6
1.0
1.4
1.8
carriers
non_carriers
GPL_acyl_acyl_58
Peak intensity x 10^{-6}
0.04
0.08
0.12
carriers
non_carriers
Ceramide_18
Peak intensity x 10^{-6}
1.0
1.5
2.0
2.5
3.0
carriers
non_carriers
Ceramide_11
Peak intensity x 10^{-6}
2
3
4
5
6
carriers
non_carriers
Ceramide_17
Peak intensity x 10^{-6}
2
3
4
5
6
7
carriers
non_carriers
Ceramide_12
Peak intensity x 10^{-6}
1.0
1.5
2.0
2.5
3.0
carriers
non_carriers
Ceramide_27
Peak intensity x 10^{-6}
0.20
0.30
0.40
0.50
carriers
non_carriers
Ceramide_04
Peak intensity x 10^{-6}
0.4
0.8
1.2
1.6
carriers
non_carriers

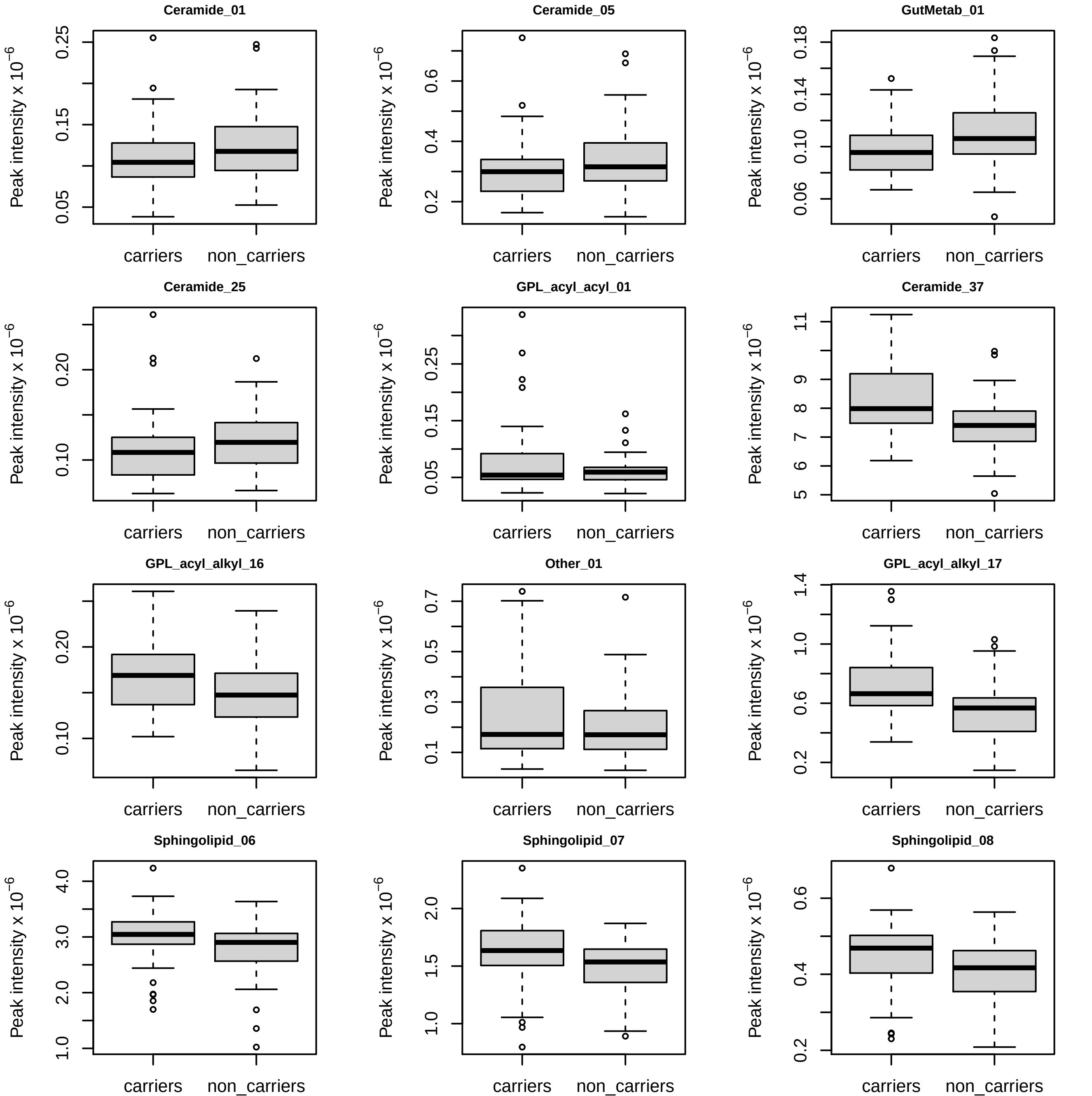
Ceramide_01
Ceramide_05
GutMetab_01
Ceramide_25
GPL_acyl_acyl_01
Ceramide_37
GPL_acyl_alkyl_16
Other_01
GPL_acyl_alkyl_17
Sphingolipid_06
Sphingolipid_07
Sphingolipid_08
Peak intensity x 10^−6
carriers
non_carriers

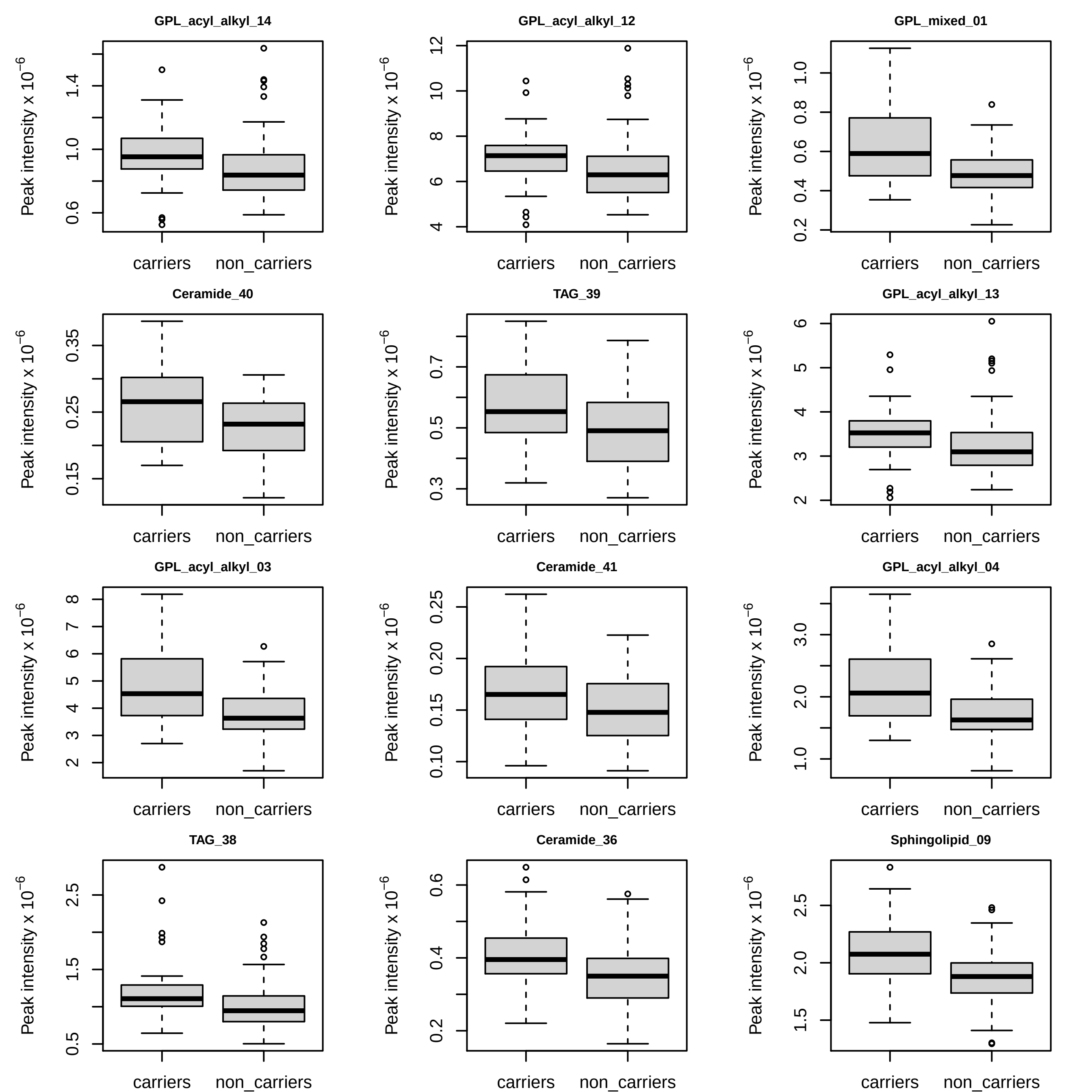

GPL_acyl_alkyl_14
GPL_acyl_alkyl_12
GPL_mixed_01
Ceramide_40
TAG_39
GPL_acyl_alkyl_13
GPL_acyl_alkyl_03
Ceramide_41
GPL_acyl_alkyl_04
TAG_38
Ceramide_36
Sphingolipid_09
Peak intensity x 10^{-6}
carriers
non_carriers

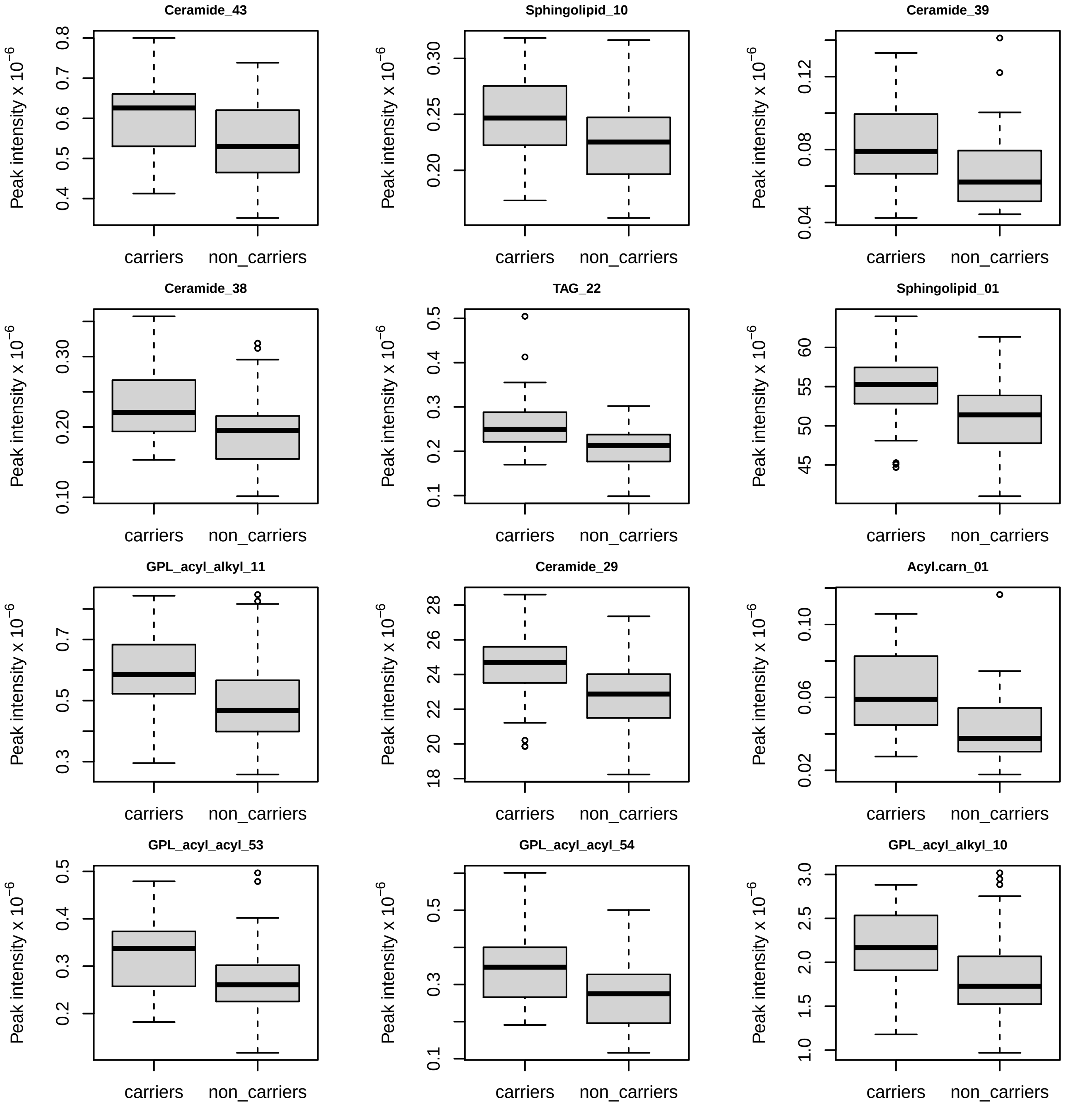
Ceramide_43
Sphingolipid_10
Ceramide_39
Ceramide_38
TAG_22
Sphingolipid_01
GPL_acyl_alkyl_11
Ceramide_29
Acyl.carn_01
GPL_acyl_acyl_53
GPL_acyl_acyl_54
GPL_acyl_alkyl_10
Peak intensity x 10^{-6}
carriers
non_carriers

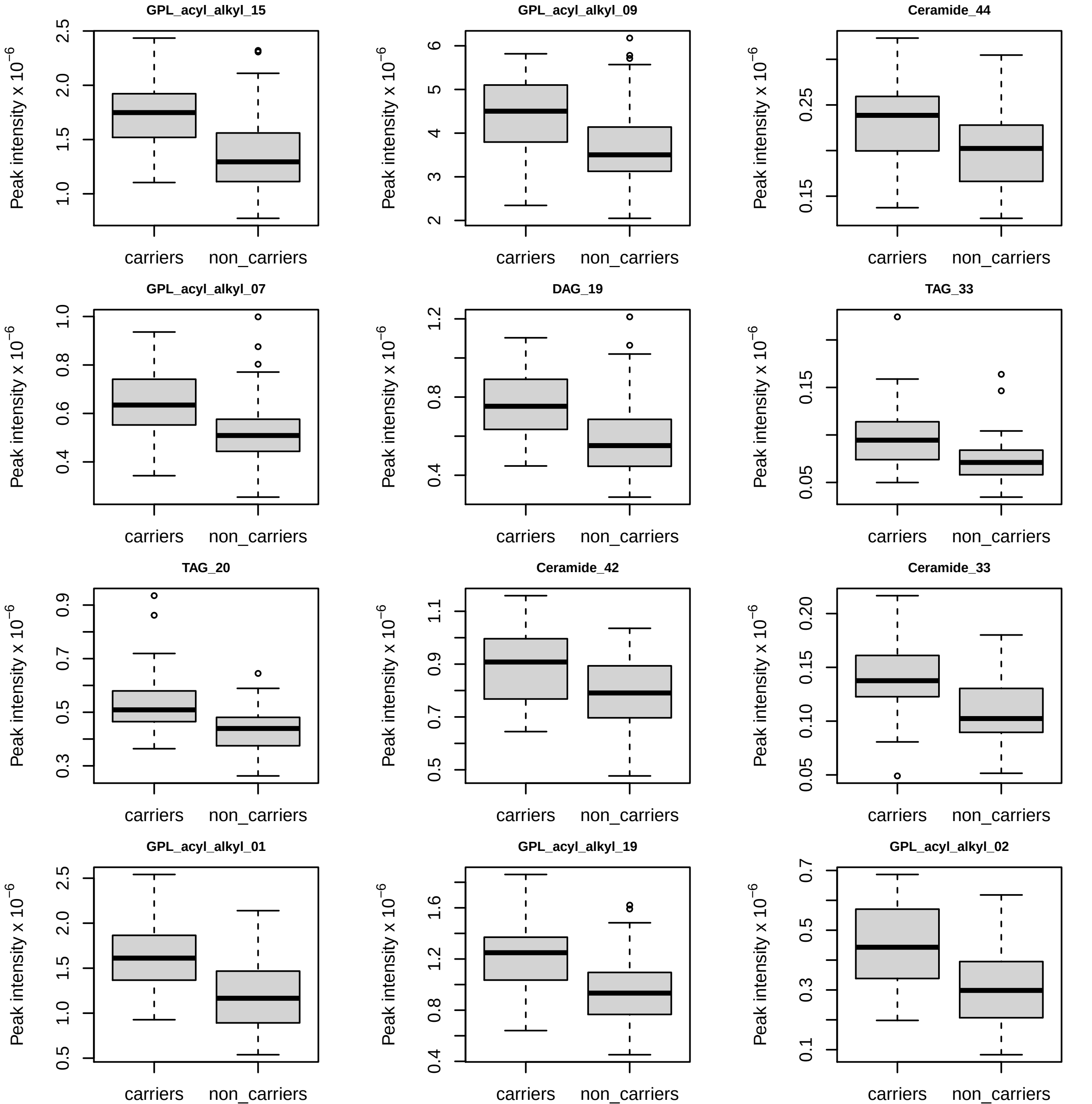
GPL_acyl_alkyl_15
GPL_acyl_alkyl_09
Ceramide_44
GPL_acyl_alkyl_07
DAG_19
TAG_33
TAG_20
Ceramide_42
Ceramide_33
GPL_acyl_alkyl_01
GPL_acyl_alkyl_19
GPL_acyl_alkyl_02
Peak intensity x 10^{-6}
carriers
non_carriers

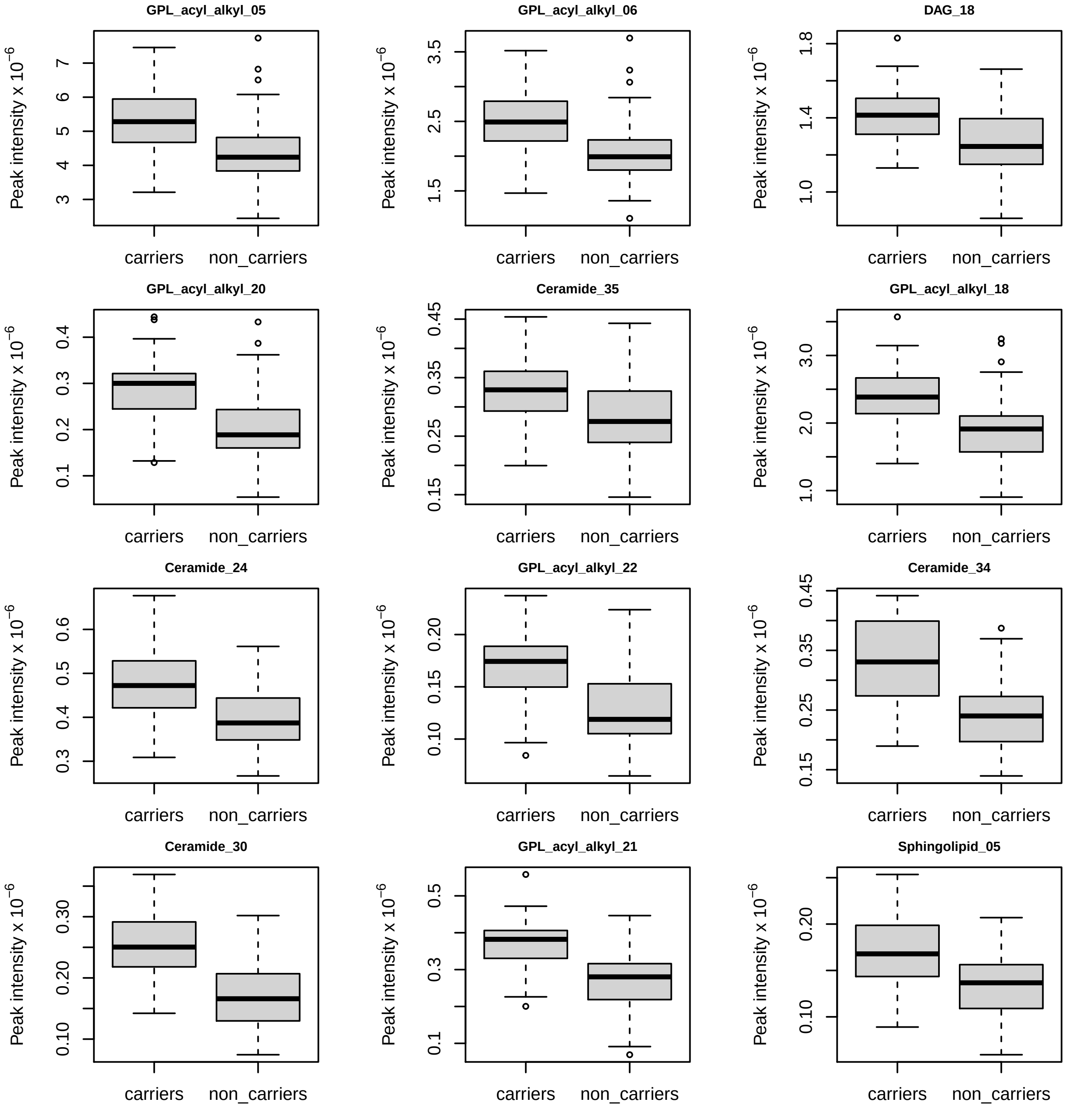

GPL_acyl_alkyl_05
Peak intensity x 10^-6
3
4
5
6
7
carriers
non_carriers
GPL_acyl_alkyl_06
Peak intensity x 10^-6
1.5
2.5
3.5
carriers
non_carriers
DAG_18
Peak intensity x 10^-6
1.0
1.4
1.8
carriers
non_carriers
GPL_acyl_alkyl_20
Peak intensity x 10^-6
0.1
0.2
0.3
0.4
carriers
non_carriers
Ceramide_35
Peak intensity x 10^-6
0.15
0.25
0.35
0.45
carriers
non_carriers
GPL_acyl_alkyl_18
Peak intensity x 10^-6
1.0
2.0
3.0
carriers
non_carriers
Ceramide_24
Peak intensity x 10^-6
0.3
0.4
0.5
0.6
carriers
non_carriers
GPL_acyl_alkyl_22
Peak intensity x 10^-6
0.10
0.15
0.20
carriers
non_carriers
Ceramide_34
Peak intensity x 10^-6
0.15
0.25
0.35
0.45
carriers
non_carriers
Ceramide_30
Peak intensity x 10^-6
0.10
0.20
0.30
carriers
non_carriers
GPL_acyl_alkyl_21
Peak intensity x 10^-6
0.1
0.3
0.5
carriers
non_carriers
Sphingolipid_05
Peak intensity x 10^-6
0.10
0.20
carriers
non_carriers

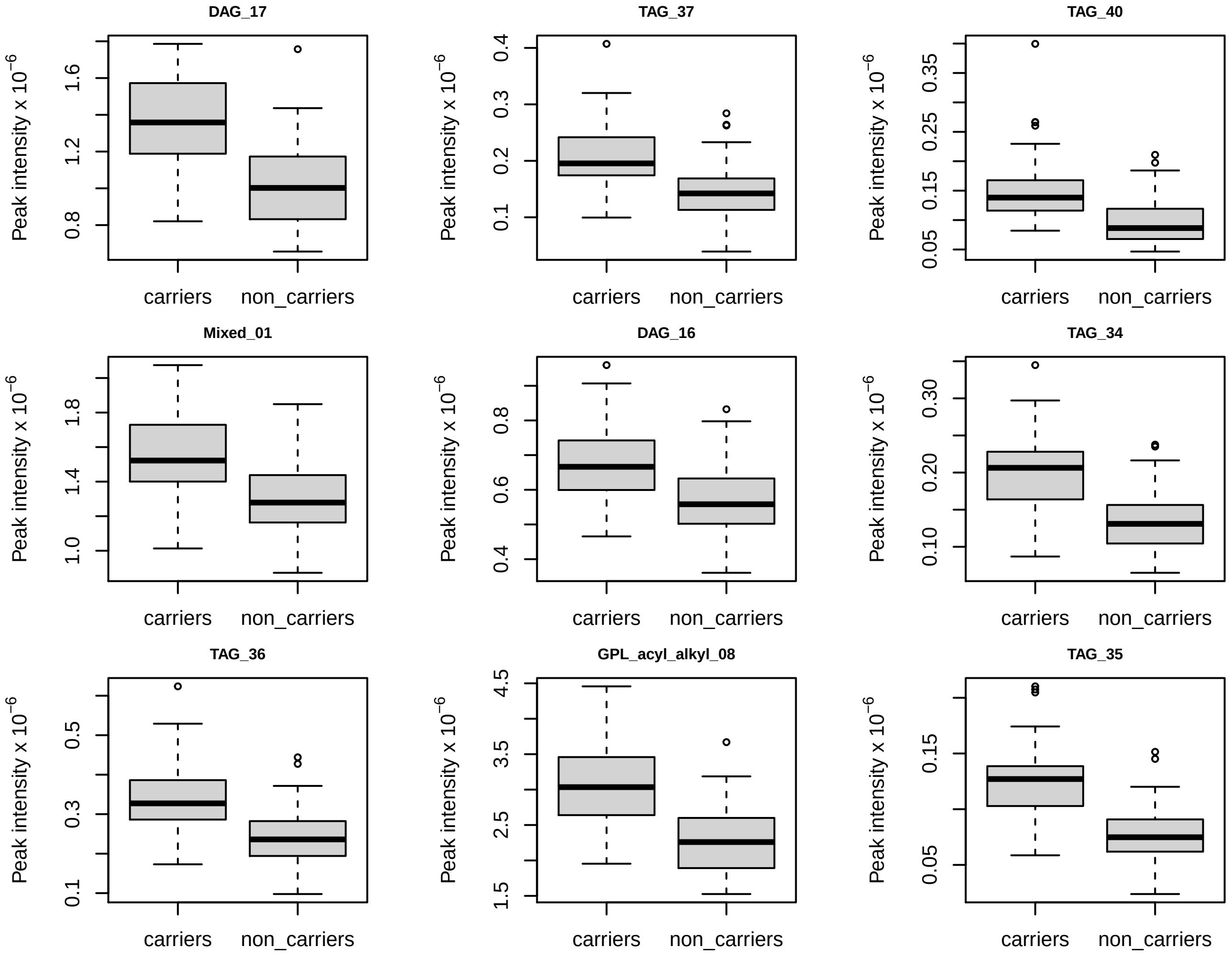

DAG_17
TAG_37
TAG_40
Mixed_01
DAG_16
TAG_34
TAG_36
GPL_acyl_alkyl_08
TAG_35
Peak intensity x 10^−6
carriers
non_carriers
0.8
1.2
1.6
0.1
0.2
0.3
0.4
0.05
0.15
0.25
0.35
1.0
1.4
1.8
0.4
0.6
0.8
0.10
0.20
0.30
0.1
0.3
0.5
1.5
2.5
3.5
4.5
0.05
0.15
